# Young rat microbiota extracts strongly inhibit fibrillation of α-synuclein and protect neuroblastoma cells and zebrafish against α-synuclein toxicity

**DOI:** 10.1016/j.mocell.2024.100161

**Published:** 2024-11-26

**Authors:** Mohaddeseh Ghorbani Shiraz, Janni Nielsen, Jeremias Widmann, Ka Hang Karen Chung, Thomas Paul Davis, Casper Rasmussen, Carsten Scavenius, Jan J. Enghild, Camille Martin-Gallausiaux, Yogesh Singh, Ibrahim Javed, Daniel E. Otzen

**Affiliations:** 1 Interdisciplinary Nanoscience Center (iNANO), Aarhus University, Gustav Wieds Vej 14, 8000 Aarhus Centrum, Denmark; 2 Australian Institute for Bioengineering and Nanotechnology, The University of Queensland, Brisbane, QLD 4072, Australia; 3 Department of Molecular Biology and Genetics, Aarhus University, Universitetsbyen 81, 8000 Aarhus Centrum, Denmark; 4 Evolutionary Biology of the Microbial Cell - Biologie Evolutive de la Cellule Microbienne Institut Pasteur, 28 Rue du Docteur Roux, Paris 75724 Cedex 15, France; 5 Institute of Medical Genetics and Applied Genomics, University of Tübingen, Calwerstraße 7, 72076 Tübingen, Germany; 6 NGS Competence Centre Tübingen (NCCT), University of Tübingen, Calwerstraße 7, 72076 Tübingen, Germany

**Keywords:** Aggregation, Microbiome, Parkinson’s disease, Proteomics, Seeding

## Abstract

The clinical manifestations of Parkinson's disease (PD) are driven by aggregation of α-Synuclein (α-Syn) in the brain. However, there is increasing evidence that PD may be initiated in the gut and thence spread to the brain, eg, via the vagus nerve. Many studies link PD to changes in the gut microbiome, and bacterial amyloid has been shown to stimulate α-Syn aggregation. Yet, we are not aware of any studies reporting on a direct connection between microbiome components and α-Syn aggregation. Here, we report that soluble extract from the gut microbiome of the rats, particularly young rats transgenic for PD, shows a remarkably strong ability to inhibit *in vitro* α-Syn aggregation and keep it natively unfolded and monomeric. The active component(s) are heat-labile molecule(s) of around 30- to 100-kDa size, which are neither nucleic acid nor lipid. Proteomic analysis identified several proteins whose concentrations in different rat samples correlated with the samples’ anti-inhibitory activity, while a subsequent pull-down assay linked the protein chaperone DnaK with the inhibitory activity of young rat’s microbiome, confirmed in subsequent *in vitro* assays. Remarkably, the microbiome extracts also protected neuroblastoma SH-SY5Y cells and zebrafish embryos against α-Syn toxicity. Our study sheds new light on the gut microbiome as a potential source of protection against PD and opens up for new microbiome-based therapeutic strategies.

## INTRODUCTION

Parkinson’s disease (PD) is a globally widespread movement disorder whose etiology is strongly linked to the aggregation of the 140-residue protein α-Synuclein (α-Syn) (Alam et al., 2019, Borghammer, 2023). One of its major clinical characteristics is the loss of dopaminergic neurons in the brain and the formation of intracellular inclusions known as Lewy bodies and Lewy neurites (Kalia and Lang, 2015). There is growing evidence that PD is not always initiated in the brain (“brain-first”) but can in some cases (“body-first”) be triggered by events in the gut or the nerves of the gastrointestinal tract, connected to the brain *via* the vagus nerve (Borghammer and Van Den Berge, 2019, Horsager et al., 2020). This is supported by the inhibitory effect of vagotomies (Svensson et al., 2015), the presence of Lewy bodies and Lewy neurites in enteric neurons (Liddle, 2018), and the disruptive effects of duodenum-inoculated α-Syn fibrils on gut function, α-Syn histopathology, and motor defects in aged mice (Challis et al., 2020). Further, in ∼80% of all PD cases, gastrointestinal dysfunction predates motor symptoms (Goldman and Postuma, 2014). There are also indications for a regulatory function of the gut microbiome on the central nervous system via the microbiota-gut-brain axis (Loh et al., 2024). This interaction involves the production of metabolites, hormones, and neurotransmitters in the gastrointestinal tract (Loh et al., 2024). Notably, fecal transplants from mice treated with 1-methyl-4-phenyl-1,2,3,6-tetrahydropyridine, a toxin linked to PD, have been shown to induce neurological deterioration (Loh et al., 2024). Finally, several case studies of fecal microbiota transplantation from healthy donors to PD patients [summarized in (Karuna et al., 2023)] report some improvement in symptoms, indicating that the microbiome composition can influence disease progression. Nevertheless, it is unresolved whether, how and which specific components in the gut can initiate (or block) α-Syn fibrillation. PD patients appear to have altered gut microbiomes compared with healthy controls (Keshavarzian et al., 2015, Scheperjans et al., 2015); genera affected include *Lactobacillus*, *Bifidobacterium, Blautia,* and *Akkermansia* (Haikal et al., 2019, Liu et al., 2024). It has been contested whether *Lactobacillus* levels increase or decrease (Unger et al., 2016, Li et al., 2019a). The controversy may reflect differences in the clinical stage at which the different samples were collected. Here, animal models have turned out to be very useful, given that gut microbiome can be monitored continuously in the knowledge that the disease will eventually develop. Thus, a very recent study, using a transgenic (TG) rat PD model (Nuber et al., 2013) that overexpresses human α-Syn under the human promoter and leads to a spatial brain distribution of α-Syn similar to endogenous human and rat expression, concluded that aging TG rats had an increased abundance of *Alistipes* and a reduction in *Lactobacillus* (Singh et al., 2023). Interestingly, these 2 phyla are considered inflammatory and anti-inflammatory, respectively. This was accompanied by an increase in levels of metabolites such as succinate, lactate, glutamate, and 4-hydroxyphenylacetate in both feces and serum (Singh et al., 2023). This is consistent with other studies showing that *Lactobacillus* is decreased in PD models and PD patients (Li et al., 2019a, Singh et al., 2019, Unger et al., 2016). The changes between wild-type (WT) and TG rats increase with age, but already at 2.5 months, there is gut bacterial dysbiosis in TG rats (Singh et al., 2023). The 2 groups of rats also differ in their behavioral phenotype, with a disruption of the olfactory discrimination already observed in young (3-month-old) TG rats (Nuber et al., 2013). At the molecular level, the TG rats accumulate human α-Syn in the colon tissue, which increases with age and reaches a plateau around 4 months (4M) (Singh et al., 2023).

It is reasonable to expect that these gut microbiome changes affect α-Syn in various ways. The intestinal bacteria are in contact with the enteric nervous system and the vagus nerve through gut enteroendocrine cells (Chandra et al., 2017, Mulak and Bonaz, 2015) and can thus transmit metabolites from the gut to the nervous system. Gut leakiness is also associated with early stages of PD in human patients (Forsyth et al., 2011) and is consistent with intestinal dysfunction in aging TG rats (Singh et al., 2023). PD rat models have also shown increased gut and systemic inflammation, as seen by increases not just in small metabolites but also different inflammatory proteins (Schwiertz et al., 2018, Singh et al., 2020). Inflammation may increase formation and aggregation of, eg*,* phosphorylated α-Syn, which is highly indicative of PD pathology (Sato et al., 2013). Besides these indirect effects, there is also the possibility of direct contact between the gut-microbial strains and α-Syn. For example, inoculation with an *Escherichia coli* strain producing the functional amyloid CsgA (curli) led to the accumulation of aggregated α-Syn not just in the gut but also in the brain, along with other physiologically detrimental changes such as microgliosis, astrogliosis, and increased brain levels of inflammatory proteins such as Toll-like receptor 2, Interleukin-6, and Tumor Necrosis Factor α (Chen et al., 2016). This is consistent with CsgA’s *in vitro* ability to stimulate α-Syn aggregation (Bhoite et al., 2022, SSampson et al., 2020). Further, the functional amyloid FapC from *Pseudomonas* also increases aggregation and deposition of the Alzheimer peptide amyloid-β (Aβ) in zebrafish, accompanied by tissue pathology and cognitive impairment (Javed et al., 2020) [conversely, monomeric Aβ can impair and reverse FapC fibrillation and accompanying biofilm formation (Ali et al., 2023)]. *Desulfovibrio* bacteria have also been shown to induce α-Syn aggregation in *Caenorhabditis elegans* (Huynh et al., 2023). Besides pathological components in the microbiome, there might also be protective factors reducing the risk of PD, such as the chaperone DnaK. As part of the Hsp70 family, DnaK is an ATP-dependent chaperone, promoting protein folding and reversing aggregation (Wawrzynów and Zylicz, 1995) by shielding hydrophobic parts from the cytosol, thus preventing unwanted interactions (Fernández-Fernández and Valpuesta, 2018). Hsp70 proteins consist of 2 domains, responsible for nucleotide and substrate binding, respectively (Fernández-Fernández and Valpuesta, 2018). ATP hydrolysis and release changes the substrate affinity, thus leading to rapid capture and release of substrate, which can even lead to active fibril disaggregation (Fernández-Fernández and Valpuesta, 2018). However, direct contact between microbiome components and α-Syn has to our knowledge not been directly investigated.

These considerations prompted us to ask a simple question: can extracts from the microbiome, specifically from TG rats disposed to PD, directly stimulate aggregation of α-Syn? Of particular interest was potential insoluble amyloid material, which might serve to cross-seed α-Syn fibrillation, given that the microbiome provides a ready source of amyloid material (Christensen et al., 2021). To test this, we collected fecal samples from specimens of the previously described rat PD TG model between the ages of 2 and 14 months (Nuber et al., 2013, Singh et al., 2023) along with its WT counterpart, prepared soluble and insoluble extracts, and tested them in a simple *in vitro* setup of α-Syn aggregation. However, the results confounded our expectations. Firstly, the extracts were highly efficient in inhibiting α-Syn aggregation rather than promoting it and maintaining α-Syn in a soluble unstructured state. Secondly, the greatest inhibitory activity was found in the soluble fraction rather than the insoluble counterpart, and inhibitory activity was the highest for young (2-month-old) rats. We present evidence that it is neither a nucleic acid nor a lipid but rather a heat-labile (and thus likely proteinaceous) component in the size range 30 to 100 kDa, which could be partially purified by gel filtration. Proteomic analysis identified DnaK as a potential fibrillation inhibitor in young rats’ microbiota. We also demonstrate that extract from *Lactobacillus*, downregulated in PD rats, has a distinct inhibitory effect, whereas the upregulated *Alistipes* has the reverse effect. Finally, we show that these promising *in vitro* properties of the rat microbiome extract are recapitulated in neuroblastoma cell lines and zebrafish embryos. In combination, our results suggest that the young gut microbiome may be a potent source of anti-PD components.

## MATERIALS AND METHODS

### Materials

Unless otherwise stated, all reagents were from Sigma-Aldrich.

### Preparation of Microbiome Extract From Rats (fecal rat microbiota)

All animal procedures were approved (TVA: HG3/18) by the authorities of the state of Baden-Württemberg, Germany. Previously, we described how overexpression of human α-Syn leads to dysregulated microbiome/metabolites with aging in a rat model of PD (Singh et al., 2023). Thus, rat fecal pellets from PD model (PD TG) and WT rats were collected at 2, 3, 6, 9, and 12 to 14 months from 2 cohorts of animals (1-9 months from cohort 1 and 12-14 months from cohort 2) to identify which time points could be critically affected by overexpression of α-Syn as described earlier (Singh et al., 2023), and were stored at −80°C. For naming, TG4 refers to rat 4 in the TG cohort etc*.* About 40 mg of each sample was transferred to a 1.5-ml Eppendorf tube, and then suspended to a 5% suspension in 0.8 ml buffer A-38 (10% sucrose and 1 mM EDTA), supplemented after suspension with 1× protease inhibitor cocktail (Roche cOmplete ULTRA Tablets, Mini, EDTA-free, 1 tab/50 ml). After 30 minutes at 37°C, the sample was centrifuged at 13,500 rpm for 15 minutes, after which the pellet was discarded. The supernatant was then centrifuged at 30,000 rpm for 1 hour at 4°C and the supernatant was stored at −20°C.

*Lactobacillus rhamnosus* (LGG ATCC 53103) and *Alistipes timonensis* (DSM 25383) were cultured for 24 hours on a shaker in aerobic and anaerobic conditions in microbial media M104 (per liter 5 g of tryptone peptone, 5 g of bacto peptone, 10 g of bacto yeast extract, 5 g of meat extract, 5 g of glucose, 2 g of NaH_2_HPO_4_, 1 ml Tween 80, 40 ml salt solution, 1 ml 0.1% reazurin, 10 ml of 0.05% hemin solution, 0.5 g of Cysteine-HCl, and 0.2 ml of liquid vitamin K1). After 24 hours, *L. rhamnosus* and *A. timonensis* had reached an OD_600_ of 1.66 and 0.76, respectively. Bacterial cultures were centrifuged at 3,000×*g* speed for 20 minutes at room temperature. After centrifugation, the culture supernatant was collected in a separate Falcon tube, filter-sterilized using 0.2-µm filter, and stored at −80°C until use.

### Fibrillation *In Vitro* Measured by ThT Fluorescence and Circular Dichroism

α-Syn was purified recombinantly in *E. coli* as described (Lorenzen et al., 2014). Fibrils were prepared by shaking 1 mg/ml α-Syn in a plate reader at 300 rpm at 37°C for 48 hours, after which the solution was spun down and the amount of fibril estimated from the amount left in the supernatant. Unless otherwise stated, all fibrillation assays were performed in a 96-well 3631 low-bind clear flat-bottomed polystyrene microplate with autoclaved glass bead. To assess the effects of microbiome extracts on fibrillation, ThT assays were performed in a total volume of 150 µl, composed of 0.3 mg/ml α-Syn monomer, 0.7% (ie, 2.1 µg/ml) preformed fibrils (PFF), 40 µM Thioflavin T, and variable amounts of microbiome extracts from rats of different ages (samples corresponding to 0.1 and 0.3 µl were diluted 10-fold to give 1 and 3 µl sample), all in PBS. When investigating the inhibitory effect of DnaK, the ThT assay was performed by coincubating 50 µM of α-Syn monomers with 0.5 µM of DnaK (Prospec Bio). Fibrillation was monitored at 37°C under shaking conditions (300 rpm) in a CLARIOstar Plus Multimode Microplate Reader (BMG Labtech) with excitation at 448 nm and emission at 485 nm. Circular dichroism (CD) spectra of these samples and their supernatants were recorded at 25°C from 280 to 190 nm on a Chirascan CD spectrometer (Applied Photophysics) in 1-nm bandwidth using a 10-nm path length. Three scans were recorded and averaged for each sample.

### Enzyme Treatment

About 5 µl from a stock of each enzyme [2 U/ml of benzonase and 1 mg/ml of *Thermomyces lanuginosus* lipase from Novozymes A/S (Rasmussen et al., 2022)] was incubated with 5 µl and 3 µl of 10× diluted fecal rat microbiota (FRaM) samples (ie, 0.5 and 0.3 µl original sample) at 37°C overnight in a total volume of 10 µl (supplemented with PBS). For heat treatment, 5 µl of 10× FRaM samples in a total volume of 10 µl were incubated for 15 minutes at 95°C. Negative controls were 5 µl PBS added to 5 µl of each of the 3 enzymes. Subsequently, these 10 µl samples were incubated with PFF and α-Syn monomer in the ThT seeding assay as described previously.

### Size Exclusion Chromatography

A Superose 6 10/300 GL column (Cytiva) was equilibrated with 1 column volume of PBS, after which 1 ml of each rat sample supernatant (2, 6, 9, and 14 months) of TG group 5 was loaded on the column separately, followed by elution with PBS while monitoring absorption at 280 nm. Fractions of 0.5 ml were collected over an ∼30 ml elution profile. A similar approach was adopted for *Lactobacillus* and *Alistipes* bacterial supernatant.

### Sample Preparation for Proteomic Analysis

Fractions 41 to 43 from size exclusion chromatography (SEC) of rat samples (2, 6, 9, and 14 months) were used. About 40 µl of each sample was lyophilized and resuspended in 18 µl 8 M urea in 100 mM ammonium bicarbonate (pH 8.0). About 2 µl 100 mM DTT was added (final concentration 10 mM), and the sample was incubated for 60 minutes. Subsequently, 2 µl 330 mM iodoacetamide was added under dim lighting (final concentration 30 mM) and the solution was incubated for 60 minutes in the dark. The solution was then quenched by adding 8.8 µl 100 mM DTT (final concentration 35 mM DTT), after which 161 µl 100 mM ammonium bicarbonate containing 2 µg/ml trypsin (Promega) was added. The solution was incubated overnight at 37°C. The tryptic peptides were purified using C18 membranes (Empore) packed into P10 pipette tips (Sarstedt). The membranes were activated with 99.9% acetonitrile and 0.1% formic acid and then equilibrated in 0.1% formic acid. The loaded peptides were washed with 0.1% formic acid, eluted with 70% acetonitrile and 0.1% formic acid. The eluted peptides were dried and subsequently dissolved in 0.1% formic acid.

### Pull-Down Assay

To identify fibril-binding proteins in the extracellular fraction, preformed α-Syn fibrils were coincubated with microbiome extracts of 2- and 14-month-old rats (prepared as previously described). About 100 µl of 1 mg/ml α-Syn fibrils in PBS were incubated with 500 µl of extract at 25°C. Incubation in 500 µl of PBS (pH 7.4) served as a negative control. All incubations were performed in triplicate. After 20 minutes, the samples were centrifuged at 22,000*g* for 15 minutes. The pellet was transferred into spin filters (Nanosep Centrifugal Filters) with a cutoff value of 300 kDa. Pellets were washed twice with 100 µl of PBS (pH 7.4) and once with 100 µl of 1 M NaCl, by incubating in the washing solution for 5 minutes followed by centrifugation at 14,000*g*. Fibrils and bound proteins were dissociated by resuspending them in 100 µl of 6 M guanidinium chloride solution for 1 hour at 25°C, followed by centrifugation. The samples were transferred into a 10-kDa MWCO filter unit, washed twice in 8 M urea, once in 8 M urea with 50 mM DTT, once in 8 M urea with 50 mM iodoacetamide, and twice in 8 M urea, with a centrifugation step at 14,000*g* for 30 minutes between each step. The unfolded, reduced, alkylated, and dried sample was digested by overnight incubation with 0.25 μg of sequencing-grade trypsin (Sigma-Aldrich) in 50 mM ammonium bicarbonate at 37°C. Following centrifugation, the pellet was suspended in 0.1% trifluoroacetic acid and micropurified utilizing P200 pipette tips (Sarstedt) filled with POROS R2 column material (Applied Biosystems) (Pudlo Nicholas et al., 2015).

### LC-MS/MS and Data Analysis

Nano liquid chromatography-mass spectrometry in tandem (LC-MS/MS) was carried out on the Orbitrap Eclipse Tribrid (Thermo Fisher Scientific) or the Q Exactive Plus Hybrid Quadrupole-Orbitrap (Thermo Fisher Scientific) mass spectrometer connected online to an EASY nanoLC 1200 (Thermo Fisher Scientific). Peptides in 0.1% formic acid were desalted on a trap column (2 cm × 100-μm inner diameter). Elution and separation were done on a 15-cm analytical column (75-µm inner diameter). The columns were packed in-house with ReproSil-Pur C18-AQ 3-µm resin (Dr. Marisch GmbH). A flow rate of 250 nl/min was used to elute the peptides with a 38-minute gradient from 6% to 44% using solvent A (0.1% formic acid) and solvent B (0.1% formic and 80% acetonitrile). The scan range was 375 to 1,500 *m/z* and the resolution of MS scans and MS/MS spectra were 70,000 and 35,000, respectively. Only ions with a charge of 2 to 5 were collected with a dynamic exclusion of 8.5 seconds.

The spectra of microbiome extract 41 to 43 were searched (November 2023) against 25 organisms listed in Supplementary Table S1. Of these, 24 were microbes that we had previously reported to constitute most of the rat microbiome (Singh et al., 2023), while the 25th organism was rat. To analyze pull-down samples, spectra were searched against human, rat, and whole bacterial phyla present in young and old rats' microbiome as reported (Singh et al., 2023). Proteome Discoverer Software (v2.5, Thermo Fisher) was used to perform label-free quantification using default settings for all analysis nodes, except where specified. Peptides were identified using the MASCOT node with trypsin allowing 2 missed cleavages. Mass tolerance was set at 10 ppm on the MS1 level, and the ion tolerance was 0.02 Da at the MS2 level. The dynamic modifications were oxidation of methionine (+15.995 Da), acetylation of the N-terminus (+42.011 Da), N-terminal loss of methionine (−131.040 Da), and combination of acetylation of the N-terminus and N-terminal loss of methionine (−89.030 Da). A static modification was defined as carbamidomethylation (+57.021 Da) on cysteines. Quantification was done on unique peptides. The MS data were further analyzed and visualized using R (v4.3.1) (R Core Team, 2013) coupled to RStudio (Team, 2015). The following packages were applied: *tidyverse* (Wickham et al., 2019), *readxl* (https://CRAN.R-project.org/package=readxl), and *ggforce* (https://CRAN.R-project.org/package=ggforce).

### Cell and Zebrafish Embryo Assays

Wild type zebrafish (*Danio rerio*) was maintained in 14:10-hour dark:light cycle in a fish circulation system at the University of Queensland Aquatic Facility at 28 ± 0.5°C, and embryos were produced by adult spawning in the morning where 2 pairs of male and female were placed in shallow water tank. The male and female were kept separated by a partition overnight and partition was removed in the morning to allow spawning. Embryos were collected after 2 hours at the bottom of the tank (Javed et al., 2019). The collected embryos were washed with Holtfreter’s buffer, containing 1% methylene blue, before microinjection. Selected zebrafish embryos (3 hours post fertilization) were microinjected with 50 nl of samples or buffer (control) in the perivitelline space chorionic fluid, of the embryos with 20 psi of pneumatic pressure using a pneumatic microinjector coupled with a manual micromanipulator (Javed et al., 2019). Sample mixture consisting of α-Syn monomers (20 µM) and 40 µM of ThT, with or without 0.1 µg/ml of microbiome extract, was prepared in sterile Holtfreter’s buffer and microinjected into the zebrafish embryos (*n* = 20 per group and 5 groups per sample). The embryos were maintained at 28 ± 0.5°C and imaged 24 hours post microinjection under green fluorescence and bright-field channel of a fluorescence microscope. The viability was assessed by the percentage of embryos that survived and developed into larvae at 24-hour time point.

Cytotoxicity assay was performed with SH-SY5Y neuroblastoma cells, where cells were cultured in a T25 flask with DMEM/F12 medium (10% fetal bovine serum and 1% PenStrep). Cells were passaged into transparent 24-well tissue-culture plates at a density of 50,000 per well and allowed to achieve ∼80% confluency. About 20 µM of α-Syn monomers, with or without 0.1 µg/ml microbiome extract, were prepared in DMEM/F12 media and added to the cells. The cells were incubated for 48 days, and the cytotoxicity and reactive oxygen species (ROS) were measured by propidium iodide (PI) and 2′,7′-dichlorofluorescein (DCF). PI and DCF at the concentration of 2 and 4 µM were added to the cells and incubated for 30 minutes. The cytotoxicity and ROS were measured by Operetta CLS High Content Analysis System (Perkin Elmer) via imaging the wells under 535 ex/615 em (PI) and 498 ex/522 em (DCF) channels. The acquired data were analyzed by Harmony software (Perkin Elmer) to calculate ROS (fluorescence intensity) and cytotoxicity (%). The internalization and aggregation of α-Syn in the cells were imaged by confocal microscopy. The α-Syn monomers were prelabeled with (10:1 molar ratio) with Alexafluor555 by 90 minutes of incubation at room conditions. The prelabeled monomers, with or without microbiome extract (2 months old, fraction 41), were added to the SH-SY5Y cells that were cultured in 18-well glass-bottom ibidi µ-Slide. The cells were incubated with samples for 48 hours, fixed in 4% paraformaldehyde (30 minutes), and permeabilized with 100 µl of 0.15% Triton X-100 in PBS. The cells were washed thrice with PBS, stained with actin-green/DAPI, and imaged for α-Syn (Alexafluor555) aggregates under a confocal laser scanning microscope.

## RESULTS

### Soluble Extract From the Microbiota of Young Rats Strongly Inhibits α-Syn Fibrillation

**Fig. 1:**
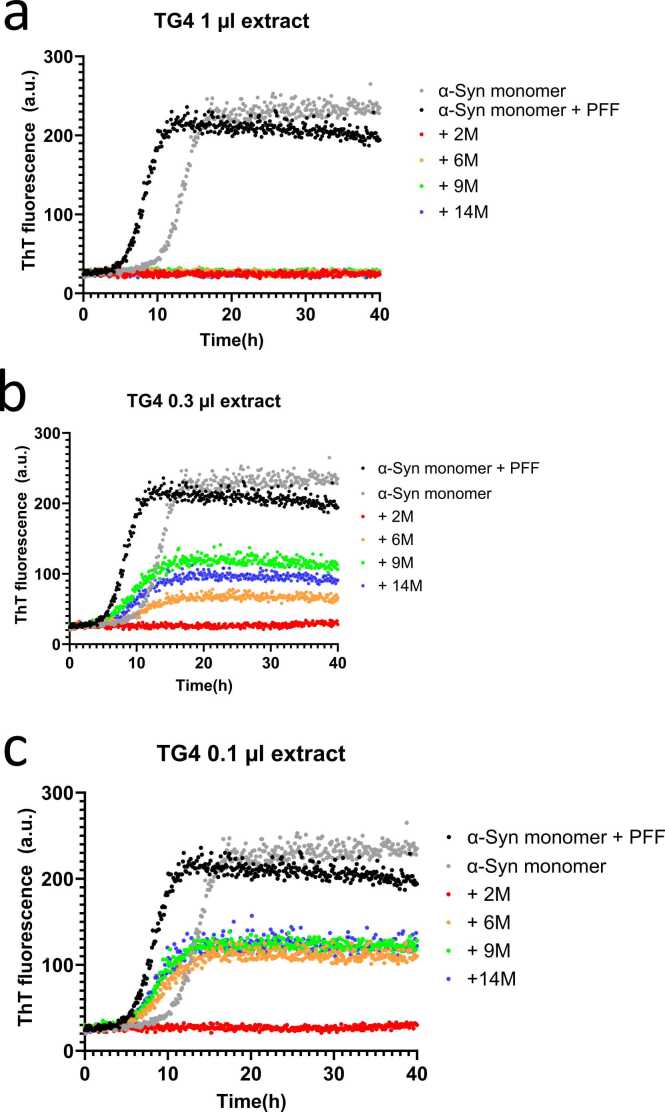
ThT time profiles of the seeded aggregation of α-Syn alone and in the presence of 0.1 to 3 µl of soluble extract of the microbiome of transgenic PD rat 4 (TG4) aged 2 to 14 months (2M-14M). Particularly the 2-month-old rats show strong inhibitory activity toward α-Syn aggregation.

The purpose of this investigation was to elucidate whether compounds present in FRaM from both PD rats (overexpressing human α-Syn) and WT rats could impact the aggregation of α-Syn. Seeded aggregation assays were carried out using monomeric α-Syn in the presence of a small (0.7% by mass) amount of preformed α-Syn fibrils (PFFs). The intention was to mimic conditions *in vivo* where it is thought that α-Syn fibrils, under some conditions, may be produced in the enteric neurons and are then transmitted from cell to cell along the vagus nerve to the brain. Particularly in leaky guts observed in some PD patients, these neurons are potentially in contact with soluble components from the intestinal fecal microbiota. Accordingly, we examined the impact of soluble extracts of FRaM on the seeded fibrillation of α-Syn. We conducted ThT assays on FRaM collected from 4 different TG PD rats and 3 WT rats at different ages (2, 6, 9, and 14 months, ie, 2M, 6M, 9M, and 14M). We chose these time points because our microbiome studies had revealed a very diverse microbiome in PD rats at 1 to 2 months, and this diversity started to reduce at later stages (3, 6, and >12 months), particularly for *Lactobacillus* strains, while there was an upsurge in *Alistipes*. This implied a switch from anti-inflammatory to inflammatory types of the microbiome, which could be initiating the disease progression (Singh et al., 2023). In the absence of FRaM, α-Syn fibrillation follows a sigmoidal time curve with a lag time of ∼6 hours, followed by a 5- to 6-h elongation or growth phase and a plateau ThT fluorescence level *ca.* 10-fold higher than the start fluorescence level (Fig. 1a). The use of a lower amount of seeds reduced the lag time by ∼5 hours, but more importantly, it firstly simulated a physiological scenario as described above and secondly led to a very robust and reproducible time course of fibrillation, allowing us to compare multiple different samples.

**Fig. 2:**
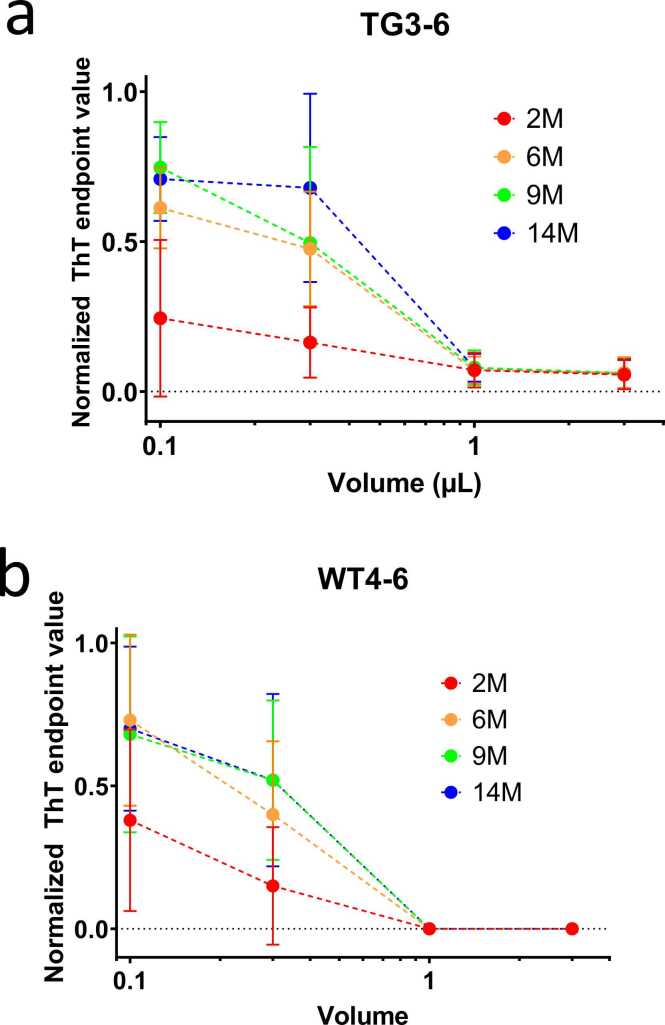
Summary of inhibitory potency of extracts from Fig. 1, measured as the averaged endpoint ThT fluorescence levels after 48 hours using extracts from (a) 4 PD rats (TG3-6) and (b) wild-type rats (WT4-6). ThT values are normalized to value in buffer without FRaM. Clearly the 2-month (2M) extracts are particularly strong at inhibiting aggregation. A similar though slightly more variable trend is seen for wild-type rats.

It was immediately clear that there was complete suppression of ThT-positive fibrillation at FRaM sample volumes as low as 1 µl per 150-µl well (corresponding to a final FRaM concentration of 0.03%, since the FRaM was already 20-fold diluted upon resuspension) (Fig. 1a). As the FRaM volume was reduced to 0.3 and 0.1 µl, some aggregation became apparent but with significant differences between samples (Fig. 1b and c). While the midpoint of fibrillation did not vary systematically between samples, there was a marked change in the endpoint ThT fluorescence levels (summarized in Fig. 2). In particular, the youngest rat samples (2M) distinguished themselves from the older ones (6M-14M) by showing close to complete suppression, even at the lowest FRaM volumes of 0.1 µl. This enhanced effect of samples from younger rats was observed for both TG PD (Fig. 1, full summary for 4 different rats in Supplementary Fig. S1) and WT (Fig. S2) rats, suggesting that it was unrelated to the overexpression of human α-Syn. We carried out analogous experiments using the insoluble fraction of the FRaM samples (see Materials and Methods) and found that the 2-month rat samples performed better than their older counterparts; however, in all cases, the effects were weaker and are most likely due to incomplete removal of soluble components from this fraction. WT rats also show some inhibitory activity, but it is slightly less consistent in its potency (Fig. 2b). This suggests for both PD and WT rats that the younger rat samples contain an inhibitory factor that suppresses α-Syn aggregation. In turn, this indicates that the composition of the rat samples changes with age and may contribute to the development or progression of neurological disorders associated with α-Syn fibrillation.

### Circular Dichroism Spectra Confirm the Lack of Aggregation of α-Syn in the Presence of Extract

**Fig. 3:**
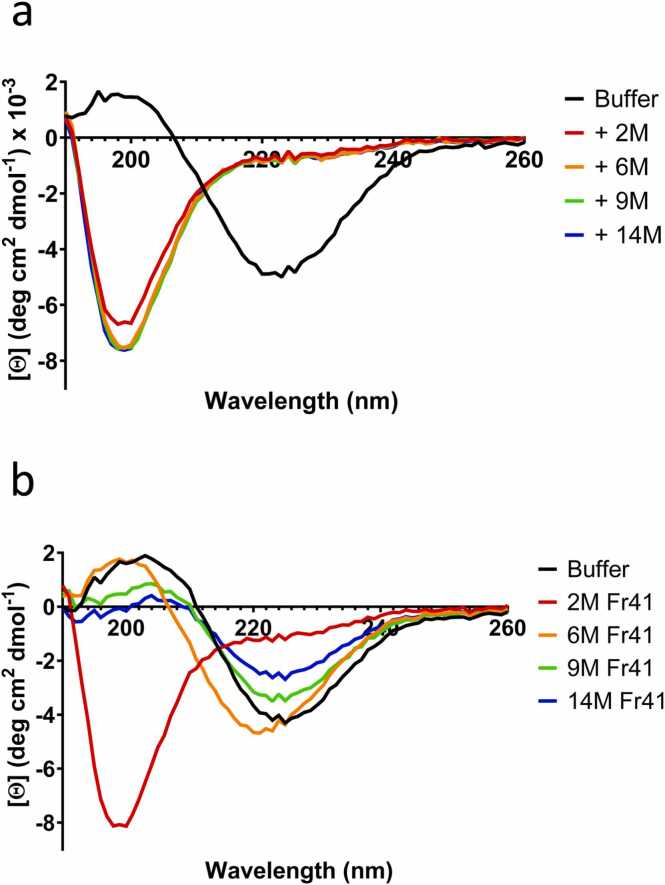
Far-UV CD spectra of α-Synuclein after aggregation in the presence of buffer (PBS) or (a) 1 µl PD TG4 rat extract of ages 2 to 14 months or (b) 10 µl of fraction 41 from SEC-fractionated TG4 FrAM of ages 2 to 14 months. There was no significant CD signal from the extracts themselves.

There is always the risk that a decrease in ThT fluorescence is an artifact caused by, eg, displacement of ThT from existing amyloid structures rather than true inhibition of aggregation (Malmos et al., 2017). To query this, we recorded far-UV CD spectra of the α-Syn samples after incubation with these FRaM samples. Monomeric α-Syn is natively unfolded with a distinct minimum around 200 nm, whereas aggregated α-Syn shows a dramatic shift in spectral appearance with a minimum around 220 nm. Gratifyingly, the resulting spectra showed unambiguously that 1 µl of all FRaM samples maintained α-Syn in a monomeric and unfolded state (just as they all suppressed ThT fluorescence in Fig. 1a), ie, the FRaM samples are true inhibitors of aggregation (Fig. 3a).

### The Aggregation-Inhibiting Species Is in the Size Range 30 to 100 kDa

**Fig. 4:**
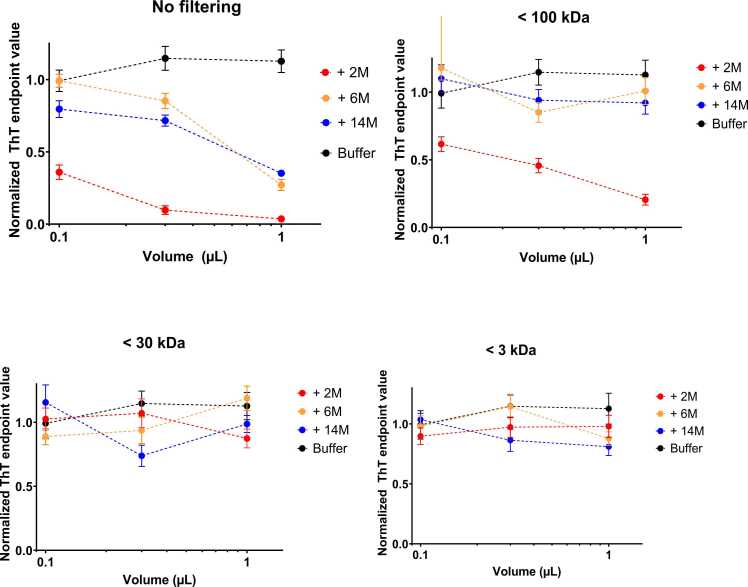
Inhibitory potency of FRaM of PD rat TG4 without size-filtering and after filtering through filters with cutoff 100, 30, and 3 kDa. Aggregation was measured from ThT assays. ThT values are normalized to value in buffer without FRaM. Error bars based on data in triplicate.

To better understand the size range of the effective component that inhibits the aggregation of α-Syn, we first filtered the original FRaM supernatant from rat sample TG4 through Amicon Ultra-0.5 centrifugal filters with cutoff sizes of 100, 30, and 3 kDa, respectively. We then conducted ThT seeding assays with 0.1 to 1 µl of the filtered samples to determine their inhibitory activity. The results were remarkably clear: whereas there was still significant inhibitory activity left in the filtrate of 2-month FRaM samples after passing through a 100-kDa cutoff, further filtration with a 30-kDa cutoff essentially removed this activity, and this was confirmed with filtration through a 3-kDa cutoff (Fig. 4). We conclude that the component(s) responsible for the inhibition of α-Syn aggregation have a size range between 100 and 30 kDa, ie, a macromolecule with a specific size and structure.

### The Aggregating Species Is Most Likely a Protein

**Fig. 5:**
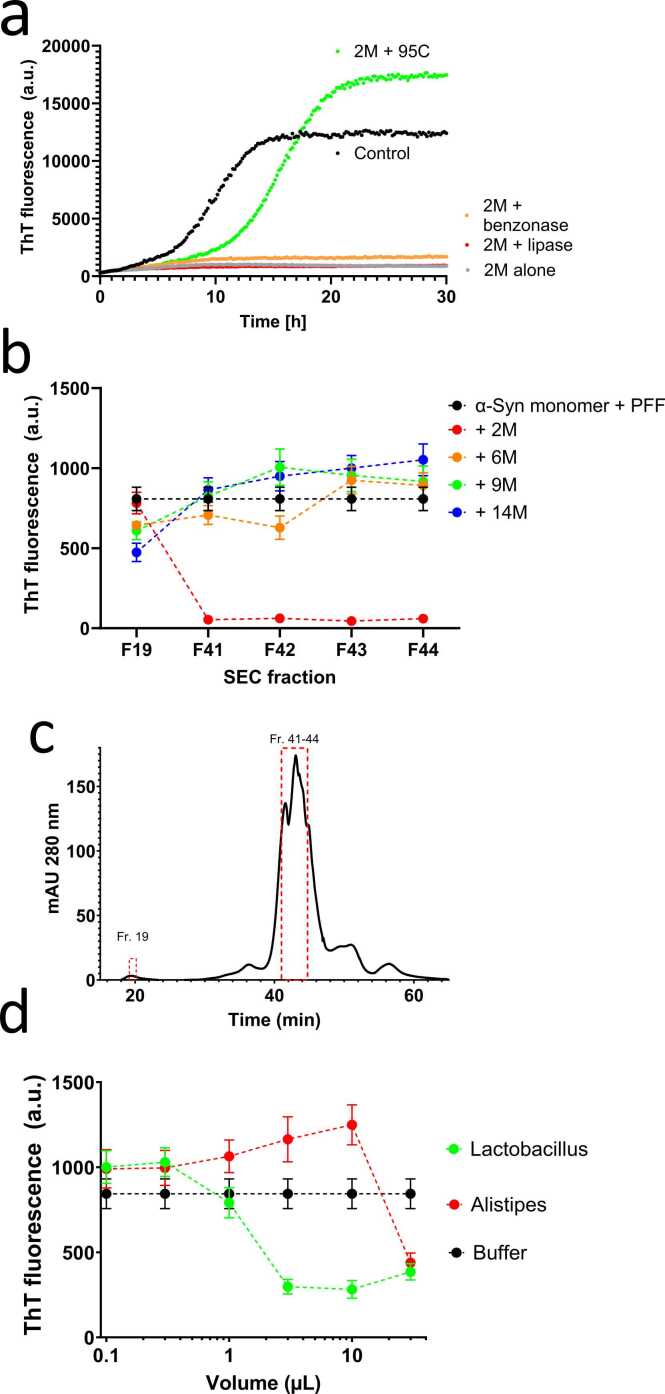
Narrowing down the active component(s) blocking α-Syn fibrillation. (a) Heat treatment but not treatment with lipase or benzonase removes inhibitory activity of 5 µl FRaM. (b) Size exclusion chromatography of the microbiome extract from 2M rats. (c) Inhibitory activity of 10 µl each of fractions 19 and 41 to 44. Data in triplicate. (d) Inhibitory activity of cell culture from *Lactobacillus* and *Alistipes*. Data in triplicate.

The next step was to identify the molecular class of this inhibitor/inhibitors by selective deactivation procedures. About 2M samples (which showed the strongest inhibitory activity) were either subjected to incubation at 95°C for 15 minutes to heat-inactivate proteins, to the promiscuous nuclease benzonase to eliminate DNA and RNA or to the lipase from *T. lanuginosus* to degrade lipids. The resulting samples were then used in ThT seeding experiments to assess their inhibitory activity (Fig. 5a). Again, the results were unambiguous: lipase and benzonase treatment did not alter the inhibitory activity of the samples, whereas heat treatment led to complete loss of inhibitory function. This finding provides strong (though still indirect) evidence that the effective component responsible for inhibiting α-Syn aggregation is neither nucleic acid nor a glycerolipid but a heat-sensitive macrobiomolecule, ie, most likely a protein.

To follow up on this, we conducted SEC to separate components based on their size. We used supernatants from 2M to 14M samples for this experiment and selected eluted fractions (monitored using absorption at 280 nm, Fig. 5b) from different size ranges from each age group and tested 2 and 10 µl of these fractions in our seeding assay. Our analysis revealed that fractions 41, 42, 43, and 44 of the 2M rat sample contained proteins that significantly inhibited α-Syn aggregation (Figs. 5c and S3), whereas the other samples did not show this remarkable inhibitory activity. Furthermore, when we compared fraction 41 from 4 different rat samples, only the 2M sample was able to maintain α-Syn in the monomeric unfolded state, whereas all other samples retained it in the fibrillated state according to circular dichroism spectroscopy (Fig. 3b), consistent with ThT data.

### Aggregatory Potency of Different Bacterial Strains Is Consistent With Microbiome Changes With Aging

Building on our previous observations that aging TG rats show a decrease in *Lactobacillus* species and an increase in *Alistipes*, we tested the inhibitory effect of 0.1 to 30 µl of supernatants obtained from spent culture media from *Lactobacillus* and *Alistipes.* Gratifyingly, *Lactobacillus* supernatant had a marked inhibitory effect on α-Syn aggregation at 3 to 30 µl, whereas *Alistipes* rather increased aggregation tendencies (Fig. 5d). However, the effects were not as strong as for our FRaM samples. Even at the highest concentrations of *Lactobacillus* sample, we only saw partial inhibition and the effect disappeared below 3 µl.

We repeated our previous crude size-separation steps using ultracentrifugation with cutoff molecular weights of 100, 30, and 3 kDa using 0.1 to 30 µl filtrate. However, unlike our previous studies, the results were less clear this time, which we attribute to the relatively weak inhibitory activity of the *Lactobacillus* supernatant (Fig. S3a-c). Nevertheless, SEC fractionation of *Lactobacillus* and *Alistipes* bacterial supernatants revealed that 50 µl of *Lactobacillus* supernatant for fractions 44, 47, 48, 51, and 52 had a somewhat greater inhibition effect on α-Syn aggregation than the corresponding fractions from *Alistipes* (Fig. S3d and e). These results suggest that the components in *Lactobacillus* supernatant have a greater potential for inhibiting α-Syn aggregation than those in *Alistipes* supernatant.

### Short-Chain Fatty Acids Do Not Affect Aggregation of α-Syn

Finally, we tested whether the small-molecule metabolites succinate, malonate, and phenylacetate, known to be formed to different extents by the microbiome under different conditions, would affect α-Syn aggregation. This was motivated by the fact that the gut microbiome produces short-chain fatty acids (SCFAs), which are upregulated in some murine PD models (Sampson et al., 2016). Several SCFAs, especially butyrate, protect against intestinal hyperpermeability and decrease inflammation through modulation of microglial activation (Huuskonen et al., 2004). Additionally, SCFA-producing bacteria are less abundant in PD patients (Vascellari et al., 2020). However, dose-response assays using 0.03 to 10 mM of these metabolites showed no significant effect on the seeding of α-Syn aggregation (data not shown).

### Proteomic Analysis of the Microbiome Samples

We carried out mass spectrometric analysis of fractions 41 to 43 from FRaM samples 2M to 14M to identify putative inhibitory proteins of α-Syn fibrillation. In total, 86 proteins were identified and peptides from 82 proteins were quantified (Fig. S4 and Supplementary Excel file MS_data_results). For some fractions, primarily fraction 41, a relatively higher abundance of certain proteins, eg, mucin 2, lipase-related protein 2, and trefoil factor 3, is seen in 2M rats, which declines in the subsequent rat samples, in the same way that the inhibitory activity of the SEC fractions declines with rat age (Fig. 5b). However, the measurements are singlets, thus statistical significance could not be calculated. Additionally, it should be noted that the chromatograms were similar with the same evenly spaced peaks (see representative chromatograms in Fig. S5). As an example, the MS1 spectra of a high-intensity peak consisted primarily of ions with a single charge (where tryptic peptides are typically minimum double-charged) in the 400- to 500-Da range. We did not attempt to identify these ions. The chromatograms showed the same pattern in all 12 measured samples and we suspect them to be contaminants.

### Identification of DnaK as a Potential Fibrillation Inhibitor in Young Rats’ Microbiota

**Fig. 6:**
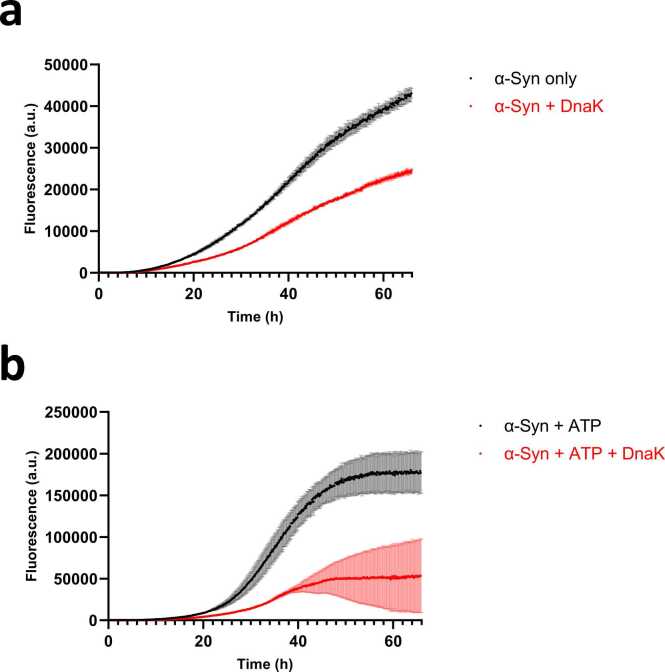
ThT time profiles of the aggregation of α-Syn alone and in the presence of DnaK in a stoichiometric ratio of 100:1 (α-Syn:DnaK). (a) Incubation of α-Syn and DnaK. (b) Incubation of α-Syn and DnaK with 2 mM ATP. DnaK shows strong inhibition of α-Syn aggregation.

As an alternative strategy to identify potential inhibitors of fibrillation, we coincubated microbiome extract with α-Syn fibrils. Inhibitors that bind stably to the high-molecular-weight fibrils were spun down with fibrils, after which the fibrils and associated proteins were dissolved and subjected to MS analysis, which identified a total of 871 different proteins. By comparing the 2- and 14-month microbiome with the negative control and selecting for gut-microbial proteins in the 2- and 14-month microbiome, the number of hits was further reduced to 328. Comparing the microbiomes of 2- and 14-month-old rats revealed 2 key differences. Firstly, the older rats’ microbiome showed increased levels of the structural protein flagellin. Secondly and more importantly, the younger rats’ microbiome had one distinct hit absent in the older cohort, which was identified as DnaK from *Blautia hydrogenotrophica*. This was encouraging for several reasons. Firstly, DnaK is already reported to inhibit α-Syn aggregation (Rodríguez-Losada et al., 2020). Secondly, *B. hydrogenotrophica* is a fecal bacterium reported to be predominantly abundant in young rats (Kakinen et al., 2021, Polanco and Götz, 2022). Moreover, the *Blautia* genus has been shown to be a discriminating feature of PD patients (Guo et al., 2022). Therefore, we investigated the closely related DnaK from *E. coli* (available commercially from Sigma-Aldrich) as a potential inhibitor in young rat’s microbiome, using a ThT assay with α-Syn monomers and DnaK. Since DnaK is an ATP-dependent chaperone (Rodríguez-Losada et al., 2020), coincubation was carried out both with and without 2 mM ATP (Fig. 6). For quantification of inhibitory effect, maximal aggregation velocities (v_max_) were calculated by linear approximation. Gratifyingly, while incubation with and without DnaK leads to the same sigmoidal growth pattern, addition of DnaK leads to a relative decrease in v_max_ (0.99 h^−1^ to 0.55 h^−1^) and end signal (43,000-24,600) by ∼45% (Fig. 6a). The addition of ATP further exacerbated this relative change in v_max_ (1.32 h^−1^ to 0.49 h^−1^) and end signal (176,900–53,000) (relative decrease ∼64%, Fig. 6b). This provided clear evidence for the inhibitory effect of DnaK on α-Syn. Furthermore, the addition of ATP led to a secondary decrease in fluorescence in 2 of the incubations, suggesting an ATP-dependent disaggregation mechanism.

### The Microbiome Samples Reduce α-Syn Aggregate Toxicity in Zebrafish Embryos and SH-SY5Y Cells

**Fig. 7:**
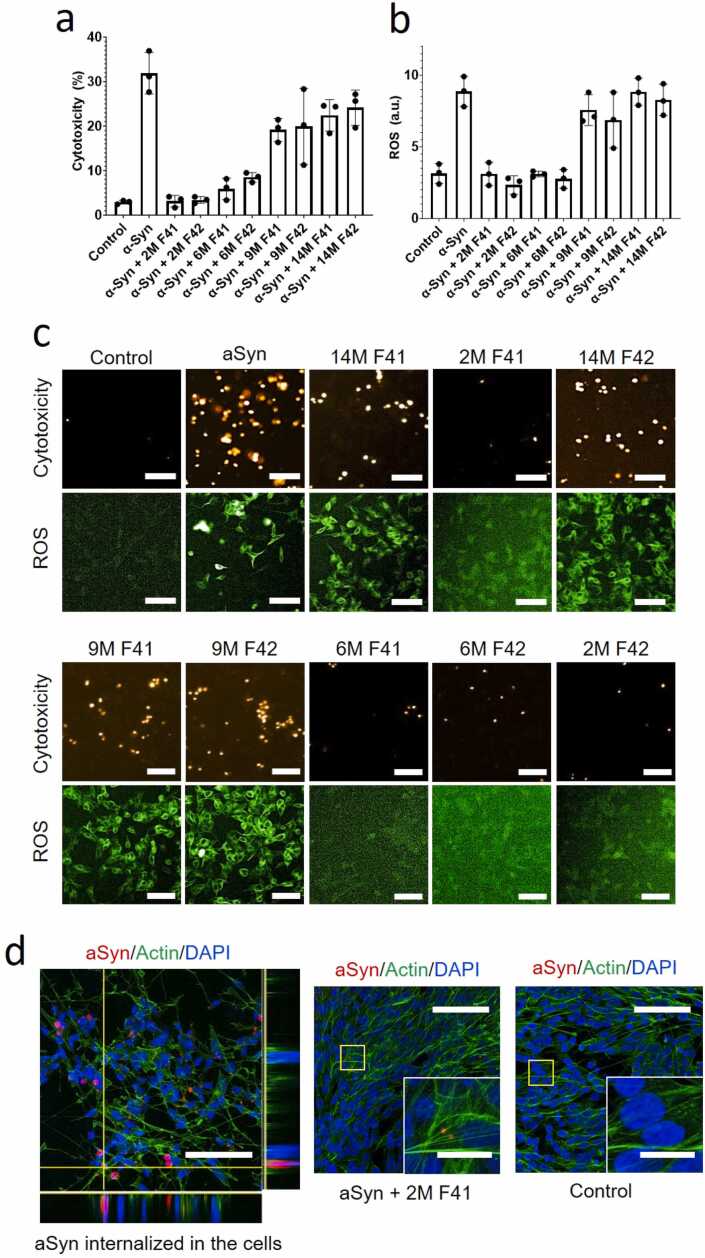
Cellular toxicity and reactive oxygen species (ROS) generation of α-Syn in SH-SY5Y neuroblastoma cells. The cytotoxicity induced by Triton-X was considered 100%, while untreated control was considered negative control (0% toxicity). Higher (a) cytotoxicity and (b) ROS were observed with α-Syn alone that was mitigated with extracts from 2- and 4-month-old rats. However, this mitigation effect was diminished in 9- and 14-month extracts. F41 and F42 represent fraction 41 and fraction 42, respectively. (c) Representative images of cytotoxicity (propidium iodide) and ROS (DCF) (scale bar: 25 µm). (d) Cellular internalization and aggregation of α-Syn in SH-SY5Y cells. Alexafluor555-labeled α-Syn monomers were incubated with the cells for 48 hours and α-Syn aggregates were internalized in the cells as imaged by confocal Z-stacking (scale bar: 25 µm). However, extract from 2M F41 inhibited the cellular interaction of α-Syn, and no intracellular α-Syn aggregates were observed, similar to untreated control (scale bar: 25 µm, insets scale bar: 10 µm). The actin and nucleus are labeled with actin-green and DAPI.

**Fig. 8:**
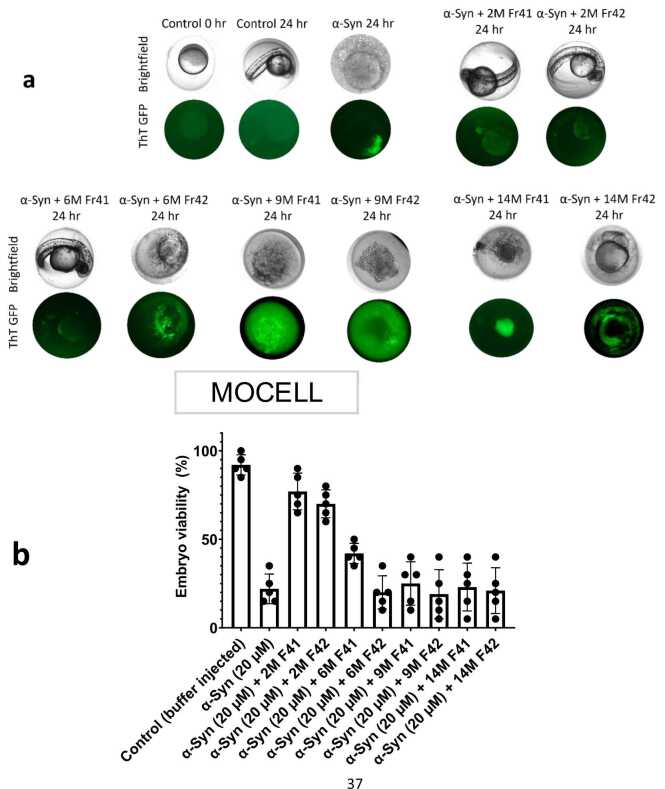
Toxicity mitigation of α-Syn by FRaM extracts in zebrafish embryonic model. (a) Representative images of zebrafish embryos in bright-field and green fluorescence channels at 24 hours post microinjection. Embryos were microinjected at 3 hours post fertilization stage (0 hour) with α-Syn, with or without FRaM extracts. Embryos developed into larvae in the absence of α-Syn toxicity or alternatively dead and imaged as embryonic debris at 24 hours post microinjection. The presence of ThT in GFP channel indicated fibrillation of α-Syn in dead embryos. Embryos were injected in groups of 5 per samples with (*n* = 20) per group. (b) The percentage of live larvae is presented as quantified embryonic viability.

Finally, to investigate the impact of these microbiome extracts in a biological setting, we tested them in 2 different systems, namely SH-SY5Y neuroblastoma cells and zebrafish embryos. SH-SY5Y cells were used as a cellular system where cells uptake α-Syn and α-Syn aggregation induces a ROS response and toxicity. SH-SY5Y neuroblastoma cells provide insight into cellular interactions and toxicity of neuropathological amyloid proteins such as Aβ, α-Syn, and Tau with both approaches of exogenous exposure and endogenous expression, in a simplified *in vitro* system (Kakinen et al., 2021, Polanco and Götz, 2022, Rodríguez-Losada et al., 2020). Zebrafish embryos provide a semi–*in vivo* and biologically complex model system where aggregation and interaction of α-Syn with embryonic cells, in the presence of biologically complex chorionic fluid, provide a toxicity model where toxicity can be mitigated by biological factors such as the microbiome. To reduce background and potential contamination effects, we use the SEC fractions 41 and 42, which showed the strongest ability to block α-Syn aggregation (Fig. 5b). In both cases, the results confirm a strong and age-dependent effect. α-Syn alone was able to induce ∼30% toxicity in the SH-SY5Y cells and elicited a substantial level of ROS (Fig. 7a-c). Extracts from 2M (and to some extent 6M) rats strongly reduce the toxicity of α-Syn in cells as well as the associated increase in ROS, while 9M and 14M extracts have little, if any effect (Fig. 7a-d). Furthermore, the 2M extract clearly blocks internalization and aggregation of α-Syn inside the cells (Fig. 7e), which suggests that the underlying mitigatory mechanism of the extract in this context is to prevent the aggregation and the physical interaction of α-Syn with the cells. Drawing on this interaction between α-Syn, cells, and extract, we microinjected α-Syn, with or without extract, into the zebrafish embryos. The zebrafish embryos provide an *in vivo* microenvironment with cells developing into larvae and any toxicity induced to those cells, in the complex medium of chorionic fluid, can impair the development of zebrafish into larvae. In zebrafish embryos, 2M extract strongly impedes the interaction of α-Syn with zebrafish embryonic cells (Fig. 8a) and mitigates α-Syn toxicity in the embryos (Fig. 8b). This effect diminished with age and was not observed in 9M and 14M extracts. The untreated embryos and those microinjected with α-Syn + 2M extract were able to develop into larvae at 24 hours post injection. α-Syn alone or in the presence of FRaM extracts from aged rats failed to mitigate α-Syn toxicity. Embryos were imaged as dead debris and ThT fluorescence indicated the presence of α-Syn fibrillation. These results corroborate the *in vitro* fibrillation assay that soluble extracts from young rats mitigate α-Syn toxicity by inhibiting its fibrillation and subsequent interaction with biological membranes.

## DISCUSSION

### The Gut Microbiome as a Potent Source of Fibrillation Inhibitors

We undertook this study in the hope that we would identify components in the gut microbiome of PD TG rats that could accelerate α-Syn fibrillation and thus provide a molecular mechanism for the transmission of disease-inducing species from the gut to the brain. This motivation was inspired by the observation that seeding the gut with curli amyloid-producing *E. coli* strains induces PD-like symptoms in rats overexpressing human α-Syn (Chen et al., 2016) and by the ability of the bacterial amyloid FapC to enhance Aβ fibrillation (Javed et al., 2020). Furthermore, the rat microbiome harbors multiple different types of amyloid, which also when expressed recombinantly readily form amyloid-like structures *in vitro* (Christensen et al., 2021). However, instead we have unexpectedly discovered that the gut microbiome of both naIve and genetically engineered PD TG rats appears to constitute a very effective reservoir of material that can suppress α-Syn aggregation *in vitro* and also protect neuronal cell line and zebrafish embryos against α-Syn toxicity. Clearly, these observations run counter to the idea that the gut microbiome only accelerates neurodegenerative processes and suggest instead an underlying protective mechanism, which, however, is lost with age and is slightly enhanced in PD rats compared with their WT counterparts. Gut microbiomes are reported to sense exogenous therapeutic molecules or endogenous mucosal glycans and respond by dynamic changes to their metabolic production (Ha et al., 2014, Pudlo Nicholas et al., 2015). Therefore, the differential anti-α-Syn activity from young PD vs WT rats can also be attributed to the feedback response of PD’s microbiome to counter the local α-Syn stress, ie, overexpression that was absent in WT rats. We are still unable to provide a satisfactory molecular explanation for these observations. However, it is worth noting that 2M rats (both PD and WT) had a higher alpha-bacterial diversity at the genera level of the gut microbiome compared with other age groups. This affects *inter alia* the bacteria *Alistipes* and *Lactobacillus* that are respectively up- and downregulated as the rats age. Consistent with this, *Alistipes* that do not protect against α-Syn fibrillation rather tended to increase, whereas *Lactobacillus* does, possibly by secreting proteins with an inhibitory effect on α-Syn fibrillation. The effects are more modest than for the original fecal samples, which likely reflects that other bacterial species may contribute as well. It is likely that aging cannot maintain this high level of different benign bacteria and thus weakens the protective power of the microbiome, leading to disease pathology. We believe that differences in the composition and efficacy of the extracted substances, depending on the age of the rats, could be decisive for regulation of α-Syn activity. Younger rats appeared to have a core competent microbiome, allowing them to suppress α-Syn aggregation. Older microbiomes, which lose different microbial strains, cannot maintain this efficacy, leading to potential disease progression. Our data indicate that this change in the composition and functional change in core microbiome impacts the production of DnaK. In addition, DnaK varies greatly between species compared with 16S rRNA (Salazar-Jaramillo et al., 2024). Thus, it is plausible that the availability and the species version of this protein change with the microbiome as we progress from younger to older rats.

### The Molecular Origins of the Protection Mechanism

We used mass spectrometry to identify, quantify, and correlate putative inhibitory proteins to α-Syn aggregation. Mass spectrometry is, in principle, an ideal method to identify such proteins, followed by validation of the impact of specific hits on α-Syn aggregation. Indeed, several proteins showed an abundance pattern consistent with the overall inhibition of aggregation, ie, high levels in the 2M sample and declining levels in the other samples. However, our results should be viewed with caution, since data could only be obtained in single replicates due to limiting amounts of sample. Furthermore, large amounts of single-charge ions were present in our samples, which dominated the chromatograms. We suspect that these single-charge ions are contaminants that caused ion suppression.

Instead, we adopted an alternative strategy of using a pull-down experiment to identify potential fibrillation inhibitors. Gratifyingly, we were able to identify DnaK from *Blautia hydrogenotrophica* as one of the specific proteins involved in this suppression of α-Syn toxicity. Its inhibitory effect was verified in a ThT assay. The difference in kinetics with and without ATP hints toward 2 separate mechanisms of inhibition. Furthermore, ATP seemed to be crucial in the disaggregatory effect of DnaK, explaining difficulties in detection with only low amounts of ATP present after washing. ATP leads to rapid release of DnaK’s substrate protein and is therefore needed for disaggregation, since the nucleotide-free state shows an even higher substrate-binding affinity (Wawrzynów and Zylicz, 1995). We believe this could explain why incubation with ATP showed disaggregation, while incubation in the absence of ATP led to an equilibrium between α-Syn amyloids and DnaK-α-Syn aggregates.

Given the precedence for cross-talk between chaperones across species and kingdoms, there may be other relevant proteins besides DnaK. Human chaperones are known to inhibit aggregation of pathological amyloid proteins such as α-Syn and Aβ and can also block aggregation of functional amyloids such as CsgA and FapC (Nagaraj et al., 2022), reflecting a generic ability to recognize aggregation-prone proteins. Furthermore, CsgC, a potent inhibitor of CsgA and FapC fibrillation (Nagaraj et al., 2022), has been reported to arrest amyloid α-Syn aggregation (Evans et al., 2015). However, our extract did not block CsgA or FapC fibrillation (data not shown), suggesting some specificity in these interactions. Perhaps, the bacterial chaperones are designed to block unwanted aggregation for which FapC and CsgA do not qualify as functional (and thus useful) amyloid. Another feasibility is the dominance of the microbiome, such as in young age vs disease or aging, by either pathogenic or symbiotic partners that can shape the overall dynamics of the microbiome’s metabolic profile (Kamada et al., 2013, Levy and Borenstein, 2013). In our case, symbiotic microbes in 2M PD rats' microbiome could result in a metabolic profile with α-Syn inhibition activity.

### Future Exploitation of α-Syn-Inhibiting Factors as Therapy Against PD

We note that the extract as well as its fractionated compounds very efficiently maintain α-Syn in the monomeric form. This requires a relatively stable physical interaction between the extract and α-Syn in order to shield it from interactions with other components. This complexation may also explain the ability of the extract to prevent the uptake of α-Syn into cells, as indicated by our cellular uptake and toxicity experiments, simply by blocking possible interaction surfaces of α-Syn with the cell membrane. The mitigation potential is lost with age, eventually resulting in α-Syn fibrillation and propagation to the brain through the enteric nervous system. Like α-Syn, Aβ also has gut presence and we have recently reported that the monomeric form of Aβ can disintegrate FapC and CsgA amyloids, dissolve the associated microbial biofilms, and reduce their cellular attachment (Ali et al., 2023). It is reasonable to hypothesize that the microbiome from young rats is dominated by symbiotic microbes that can stabilize peptides and proteins such as Aβ or α-Syn in the gut for their physiological roles.

Naturally, it would be highly interesting to investigate possible parallels in the development of the human gut microbiome, eg, a comparative study of its anti-aggregatory potency at different host ages. However, this is clearly a major undertaking with significant logistical challenges and many confounding factors. Here, the short life span of the rat and the possibility to compare WT and PD strains with a known and expected development of symptoms under highly controlled living conditions is a significant advantage. For example, controlled fecal microbiome transplantations between young and old rats may help establish the full scope of such an intervention. More importantly, studies of the rat gut microbiome may provide sufficient new insights for therapeutic purposes. Thus, we simply need to be able to provide DnaK and other relevant proteins on a regular basis. This will make us independent of any gradual decline in the population of benign bacteria and thus develop more effective strategies to proactively protect against the development of gut-first PD. One option would be to overexpress the proteins in question in *E. coli* strains designed to effectively colonize the gut. A good candidate is the *E. coli* Nissle strain, which is anchored to the gut lining using the mucoadhesive trefoil factor (Praveschotinunt et al., 2019) (interestingly enough one of the proteins found to be present to a higher extent in the 2M samples than the other samples). The engineered strain effectively treated colitis in mice, restoring a healthy phenotype within 5 days (Praveschotinunt et al., 2019). In addition, the mutant strain did not show increased pathogenicity compared with the WT (Praveschotinunt et al., 2019). Therefore, replacing the mucoadhesive trefoil factor with DnaK or other benign protein inhibitors of α-Syn aggregation may be a viable strategy for preventing gut-originating PD, by disaggregating fibrils to prevent penetration through the mucosal membrane.

## Author Contributions

Casper Rasmussen: Investigation, formal analysis. Carsten Scavenius: Investigation, formal analysis. Jan J. Enghild: Supervision. Camille Martin-Gallausiaux: Investigation. Yogesh Singh: Writing—review and editing, resources, and conceptualization. Ibrahim Javed: Writing—review and editing, supervision, investigation, and formal analysis. Daniel E. Otzen: Writing—review and editing, writing—original draft, supervision, resources, project administration, methodology, investigation, funding acquisition, formal analysis, and conceptualization. Mohaddeseh Ghorbani Shiraz: Investigation. Janni Nielsen: Investigation. Jeremias Widmann: Investigation, formal analysis. Ka Chung: Investigation. Thomas Davis: Investigation.

## Declaration of Competing Interests

The authors declare that they have no known competing financial interests or personal relationships that could have appeared to influence the work reported in this paper.

## References

[bib1] Alam P., Bousset L., Melki R., Otzen D.E. (2019). Alpha-synuclein oligomers and fibrils: a spectrum of species, a spectrum of toxicities. J. Neurochem..

[bib2] Ali S.A., Chung K.H.K., Forgham H., Kakinen A., Olsen W.P., Balaji A., Otzen D.E., Davis T.P., Javed I. (2023). Alzheimer’s progenitor amyloid-β targets and dissolves microbial amyloids and impairs biofilm function. Adv. Sci..

[bib3] Bhoite S.S., Han Y., Ruotolo B.T., Chapman M.R. (2022). Mechanistic insights into accelerated α-synuclein aggregation mediated by human microbiome-associated functional amyloids. J. Biol. Chem..

[bib4] Borghammer P. (2023). The brain-first vs. body-first model of Parkinson's disease with comparison to alternative models. J. Neural Transm. (Vienna).

[bib5] Borghammer P., Van Den Berge N. (2019). Brain-first versus gut-first Parkinson's disease: a hypothesis. J. Parkinsons Dis..

[bib6] Challis C., Hori A., Sampson T.R., Yoo B.B., Challis R.C., Hamilton A.M., Mazmanian S.K., Volpicelli-Daley L.A., Gradinaru V. (2020). Gut-seeded α-synuclein fibrils promote gut dysfunction and brain pathology specifically in aged mice. Nat. Neurosci..

[bib7] Chandra R., Hiniker A., Kuo Y.-M., Nussbaum R.L., Liddle R.A. (2017). α-Synuclein in gut endocrine cells and its implications for Parkinson’s disease. *JCI Insight*.

[bib8] Chen S.G., Stribinskis V., Rane M.J., Demuth D.R., Gozal E., Roberts A.M., Jagadapillai R., Liu R., Choe K., Shivakumar B. (2016). Exposure to the functional bacterial amyloid protein curli enhances alpha-synuclein aggregation in aged Fischer 344 rats and *Caenorhabditis elegans*. Sci. Rep..

[bib9] Christensen L.F.B., Alijanvand S.H., Burdukiewicz M., Herbst F.A., Kjeldal H., Dueholm M.S., Otzen D.E. (2021). Identification of amyloidogenic proteins in the microbiomes of a rat Parkinson's disease model and wild-type rats. Protein Sci..

[bib10] Evans M.L., Chorell E., Taylor J.D., Aden J., Gotheson A., Li F., Koch M., Sefer L., Matthews S.J., Wittung-Stafshede P. (2015). The bacterial curli system possesses a potent and selective inhibitor of amyloid formation. Mol. Cell.

[bib11] Forsyth C.B., Shannon K.M., Kordower J.H., Voigt R.M., Shaikh M., Jaglin J.A., Estes J.D., Dodiya H.B., Keshavarzian A. (2011). Increased intestinal permeability correlates with sigmoid mucosa alpha-synuclein staining and endotoxin exposure markers in early Parkinson's disease. PLoS One.

[bib12] Haikal C., Chen Q.-Q., Li J.-Y. (2019). Microbiome changes: an indicator of Parkinson’s disease?. Transl. Neurodegener..

[bib13] Goldman J.G., Postuma R. (2014). Premotor and nonmotor features of Parkinson’s disease. Curr. Opin. Neurol..

[bib14] Guo X., Tang P., Hou C., Chong L., Zhang X., Liu P., Chen L., Liu Y., Zhang L., Li R. (2022). Integrated microbiome and host transcriptome profiles link Parkinson’s disease to blautia genus: evidence from feces, blood, and brain. Front. Microbiol..

[bib15] Ha C.W., Lam Y.Y., Holmes A.J. (2014). Mechanistic links between gut microbial community dynamics, microbial functions and metabolic health. World J. Gastroenterol..

[bib16] Horsager J., Andersen K.B., Knudsen K., Skjærbæk C., Fedorova T.D., Okkels N., Schaeffer E., Bonkat S.K., Geday J., Otto M. (2020). Brain-first versus body-first Parkinson's disease: a multimodal imaging case-control study. Brain.

[bib17] Huuskonen J., Suuronen T., Nuutinen T., Kyrylenko S., Salminen A. (2004). Regulation of microglial inflammatory response by sodium butyrate and short-chain fatty acids. Br. J. Pharmacol..

[bib18] Huynh V.A., Takala T.M., Murros K.E., Diwedi B., Saris P.E.J. (2023). Desulfovibrio bacteria enhance alpha-synuclein aggregation in a *Caenorhabditis elegans* model of Parkinson’s disease. Front. Cell. Infect. Microbiol..

[bib19] Javed I., Peng G., Xing Y., Yu T., Zhao M., Kakinen A., Faridi A., Parish C.L., Ding F., Davis T.P. (2019). Inhibition of amyloid beta toxicity in zebrafish with a chaperone-gold nanoparticle dual strategy. Nat. Commun..

[bib20] Javed I., Zhang Z., Adamcik J., Andrikopoulos N., Li Y., Otzen D.E., Lin S., Mezzenga R., Davis T.P., Ding F. (2020). Accelerated amyloid beta pathogenesis by bacterial amyloid FapC. Adv Sci (Weinh).

[bib21] Kakinen A., Javed I., Davis T.P., Ke P.C. (2021). In vitro and in vivo models for anti-amyloidosis nanomedicines. Nanoscale Horiz..

[bib22] Kalia L.V., Lang A.E. (2015). Parkinson's disease. Lancet.

[bib23] Kamada N., Chen G.Y., Inohara N., Núñez G. (2013). Control of pathogens and pathobionts by the gut microbiota. Nat. Immunol..

[bib24] Karuna E.W.V., Vlada O.C., Ed J.K., Elisabeth M.T., Jacobus Jv.H., Maria Fiorella C. (2023). Safety and feasibility of faecal microbiota transplantation for patients with Parkinson’s disease: a protocol for a self-controlled interventional donor-FMT pilot study. BMJ Open.

[bib25] Keshavarzian A., Green S.J., Engen P.A., Voigt R.M., Naqib A., Forsyth C.B., Mutlu E., Shannon K.M. (2015). Colonic bacterial composition in Parkinson's disease. Mov. Disord..

[bib26] Levy R., Borenstein E. (2013). Metabolic modeling of species interaction in the human microbiome elucidates community-level assembly rules. Proc. Natl. Acad. Sci..

[bib27] Liddle R.A. (2018). Parkinson’s disease from the gut. Brain Res..

[bib28] Liu J., Lv X., Ye T., Zhao M., Chen Z., Zhang Y., Yang W., Xie H., Zhan L., Chen L. (2024). Microbiota-microglia crosstalk between *Blautia producta* and neuroinflammation of Parkinson's disease: a bench-to-bedside translational approach. Brain Behav. Immun..

[bib29] Li C., Cui L., Yang Y., Miao J., Zhao X., Zhang J., Cui G., Zhang Y. (2019). Gut microbiota differs between Parkinson’s disease patients and healthy controls in Northeast China. Front. Mol. Neurosci..

[bib30] Loh J.S., Mak W.Q., Tan L.K.S., Ng C.X., Chan H.H., Yeow S.H., Foo J.B., Ong Y.S., How C.W., Khaw K.Y. (2024). Microbiota–gut–brain axis and its therapeutic applications in neurodegenerative diseases. Signal Transduct. Target. Ther..

[bib31] Lorenzen N., Nielsen S.B., Buell A.K., Kaspersen J.D., Arosio P., Vad B.S., Paslawski W., Christiansen G., Valnickova-Hansen Z., Andreasen M. (2014). The rol me of stable α-synuclein oligomers in the molecular events underlying amyloid formation. J. Am. Chem. Soc..

[bib32] Malmos K.G., Blancas-Mejia L., Weber B., Buchner J., Ramirez-Alvarado M., Naiki H., Otzen D.E. (2017). ThT 101: a primer on the use of Thioflavin T to investigate amyloid formation. Amyloid.

[bib33] Mulak A., Bonaz B. (2015). Brain-gut-microbiota axis in Parkinson's disease. World J. Gastroenterol..

[bib34] Nagaraj M., Najarzadeh Z., Pansieri J., Biverstål H., Musteikyte G., Smirnovas V., Matthews S., Emanuelsson C., Johansson J., Buxbaum J.N. (2022). Chaperones mainly suppress primary nucleation during formation of functional amyloid required for bacterial biofilm formation. Chem. Sci..

[bib35] Nuber S., Harmuth F., Kohl Z., Adame A., Trejo M., Schonig K., Zimmermann F., Bauer C., Casadei N., Giel C. (2013). A progressive dopaminergic phenotype associated with neurotoxic conversion of alpha-synuclein in BAC-transgenic rats. Brain.

[bib36] Polanco J.C., Götz J. (2022). Exosomal and vesicle-free tau seeds—propagation and convergence in endolysosomal permeabilization. FEBS J..

[bib37] Praveschotinunt P., Duraj-Thatte A.M., Gelfat I., Bahl F., Chou D.B., Joshi N.S. (2019). Engineered *E. coli* Nissle 1917 for the delivery of matrix-tethered therapeutic domains to the gut. Nat. Commun..

[bib38] Pudlo Nicholas A., Urs K., Kumar Supriya S., German J.B., Mills D.A., Martens Eric C. (2015). Symbiotic human gut bacteria with variable metabolic priorities for host mucosal glycans. mBio.

[bib39] Fernández-Fernández M.R., Valpuesta J.M. (2018). Hsp70 chaperone: a master player in protein homeostasis. F1000Research.

[bib40] R Core Team. (2013). R: A language and environment for statistical computing.

[bib41] Rasmussen, H O., Wollenberg D.T.W., Wang H., Andersen K.K., Oliveira C.L.P., Jorgensen C.I., Jorgensen T.J.D., Otzen D.E., Pedersen J.S. (2022). The changing face of SDS denaturation: complexes of *Thermomyces lanuginosus* lipase with SDS at pH 4.0, 6.0 and 8.0. J. Colloid Interface Sci..

[bib42] Rodríguez-Losada N., de la Rosa J., Larriva M., Wendelbo R., Aguirre J.A., Castresana J.S., Ballaz S.J. (2020). Overexpression of alpha-synuclein promotes both cell proliferation and cell toxicity in human SH-SY5Y neuroblastoma cells. J. Adv. Res..

[bib43] Salazar-Jaramillo L., de la Cuesta-Zuluaga J., Chica Luis A., Cadavid M., Ley Ruth E., Reyes A., Escobar Juan S. (2024). Gut microbiome diversity within Clostridia is negatively associated with human obesity. mSystems.

[bib44] Sampson T.R., Challis C., Jain N., Moiseyenko A., Ladinsky M.S., Shastri G.G., Thron T., Needham B.D., Horvath I., Debelius J.W. (2020). A gut bacterial amyloid promotes a-synuclein aggregation and motor impairment in mice. eLife.

[bib45] Sampson T.R., Debelius J.W., Thron T., Janssen S., Shastri G.G., Ilhan Z.E., Challis G.G., Schretter C.E., Rocha S., Gradinaru V. (2016). Gut microbiota regulate motor deficits and neuroinflammation in a model of Parkinson's disease. Cell.

[bib46] Sato H., Kato T., Arawaka S. (2013). The role of Ser129 phosphorylation of alpha-synuclein in neurodegeneration of Parkinson's disease: a review of in vivo models. Rev. Neurosci..

[bib47] Scheperjans F., Aho V., Pereira P.A.B., Koskinen K., Paulin L., Pekkonen E., Haapaniemi E., Kaakkola S., Eerola-Rautio J., Pohja M. (2015). Gut microbiota are related to Parkinson's disease and clinical phenotype. Mov. Disord..

[bib48] Schwiertz A., Spiegel J., Dillmann U., Grundmann D., Bürmann J., Faßbender K., Schäfer K.-H., Unger M.M. (2018). Fecal markers of intestinal inflammation and intestinal permeability are elevated in Parkinson's disease. Parkinsonism Relat. Disord..

[bib49] Singh Y., El-Hadidi M., Admard J., Wassouf Z., Schulze-Hentrich J.M., Kohlhofer U., Quintanilla-Martinez L., Huson D., Riess O., Casadei N. (2019). Enriched environmental conditions modify the gut microbiome composition and fecal markers of inflammation in Parkinson's disease. Front. Neurosci..

[bib50] Singh Y., Trautwein C., Dhariwal A., Salker M.S., Alauddin M., Zizmare L., Pelzl L., Feger M., Admard J., Casadei N. (2020). DJ-1 (Park7) affects the gut microbiome, metabolites and the development of innate lymphoid cells (ILCs). Sci. Rep..

[bib51] Singh Y., Trautwein C., Romani J., Salker M.S., Neckel P.H., Fraccaroli I., Abeditashi M., Woerner N., Admard J., Dhariwal A. (2023). Overexpression of human alpha-Synuclein leads to dysregulated microbiome/metabolites with ageing in a rat model of Parkinson disease. Mol. Neurodegener..

[bib52] Svensson E., Horvath-Puho E., Thomsen R.W., Djurhuus J.C., Pedersen L., Borghammer P., Sorensen H.T. (2015). Vagotomy and subsequent risk of Parkinson's disease. Ann. Neurol..

[bib53] Team R. (2015). RStudio: integrated development for R.

[bib54] Unger M.M., Spiegel J., Dillmann K.U., Grundmann D., Philippeit H., Burmann J., Fassbender K., Schwiertz A., Schafer K.H. (2016). Short chain fatty acids and gut microbiota differ between patients with Parkinson's disease and age-matched controls. Parkinsonism Relat. Disord..

[bib55] Vascellari S., Palmas V., Melis M., Pisanu S., Cusano R., Uva P, Perra D., Madau V., Sarchioto M., Oppo V. (2020). Gut Microbiota and metabolome alterations associated with Parkinson’s disease. mSystems.

[bib56] Wawrzynów A., Zylicz M. (1995). Divergent effects of ATP on the binding of the DnaK and DnaJ chaperones to each other, or to their various native and denatured protein substrates. J. Biol. Chem..

[bib57] Wickham H., Averick M., Bryan J., Chang W.S., McGowan L., Francois R., Grolemund G., Hayes A., Henry L., Hester J. (2019). Welcome to the Tidyverse. J. Open Source Softw..

