# Supplementary material for: Young rat microbiota extracts strongly inhibit fibrillation of α-synuclein and protect neuroblastoma cells and zebrafish against α-synuclein toxicity
Source: Mol Cells. 2024 Nov 26;48(1):100161. doi: 10.1016/j.mocell.2024.100161 (PMC11699742; doi:10.1016/j.mocell.2024.100161)
Supplement: Supplementary file 1 — Supplementary material [file mmc1.pptx]

## Slide 1
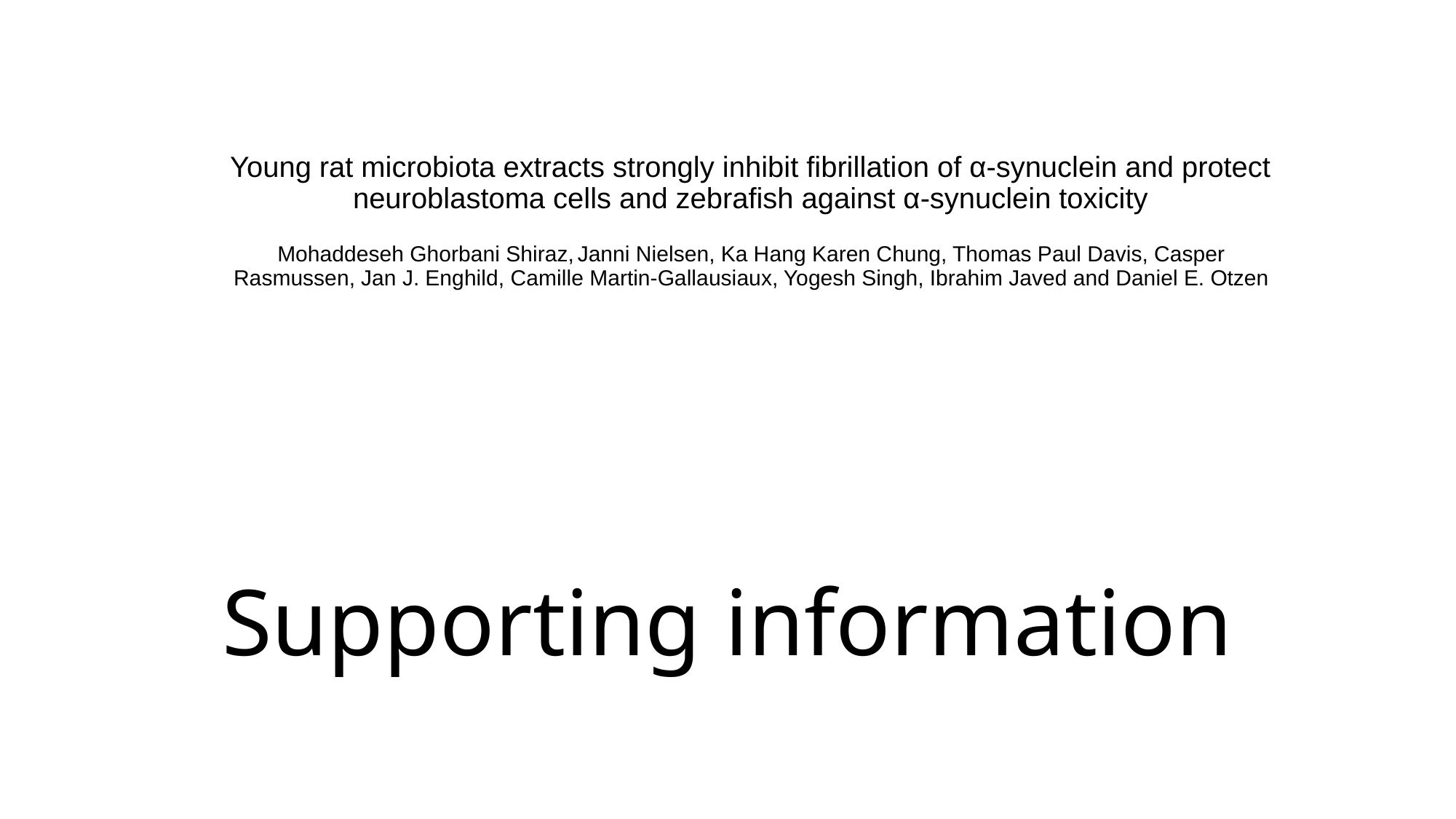

Young rat microbiota extracts strongly inhibit fibrillation of α-synuclein and protect neuroblastoma cells and zebrafish against α-synuclein toxicity
Mohaddeseh Ghorbani Shiraz, Janni Nielsen, Ka Hang Karen Chung, Thomas Paul Davis, Casper Rasmussen, Jan J. Enghild, Camille Martin-Gallausiaux, Yogesh Singh, Ibrahim Javed and Daniel E. Otzen
# Supporting information

## Slide 2
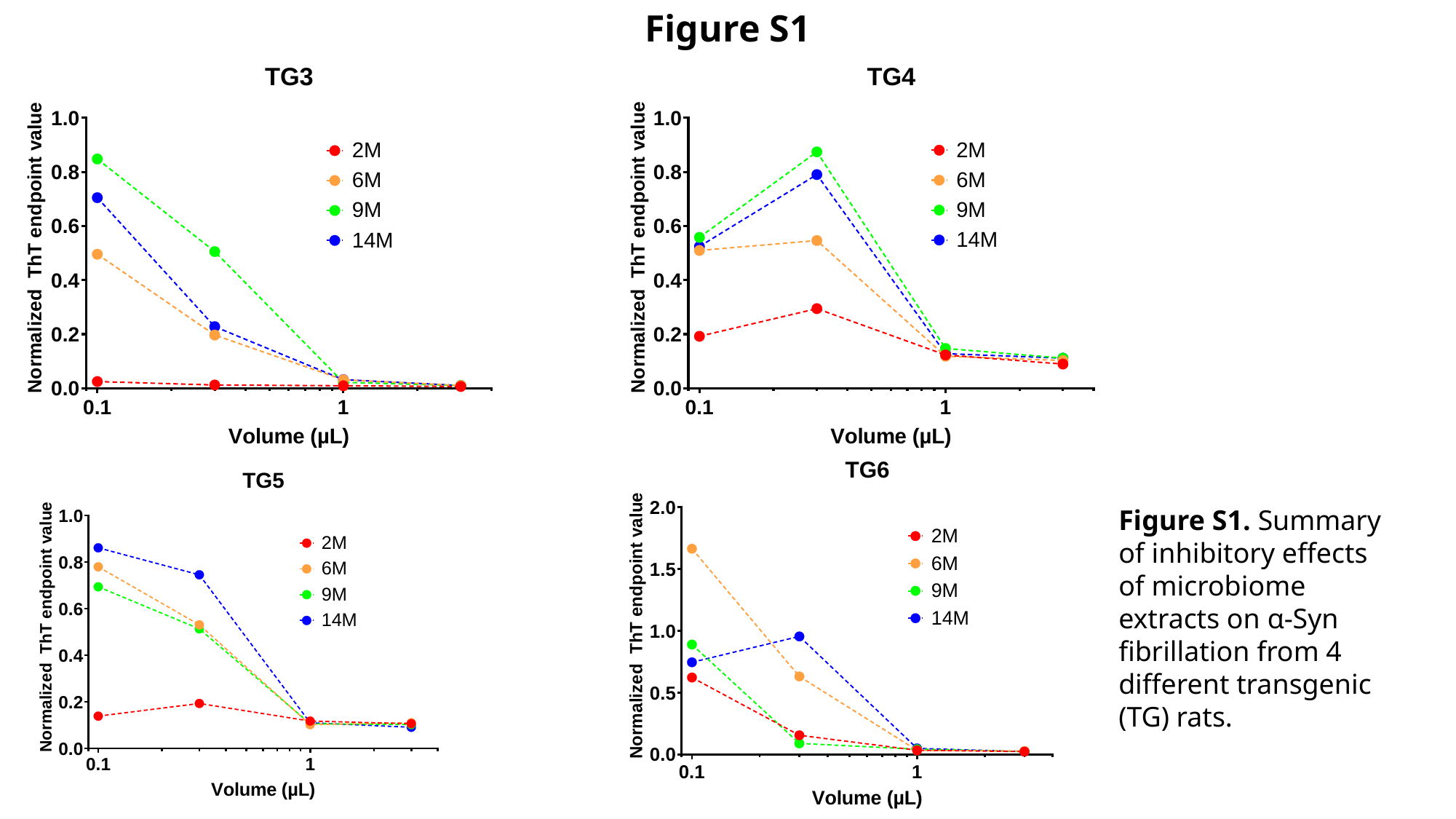

Figure S1
Figure S1. Summary of inhibitory effects of microbiome extracts on α-Syn fibrillation from 4 different transgenic (TG) rats.

## Slide 3
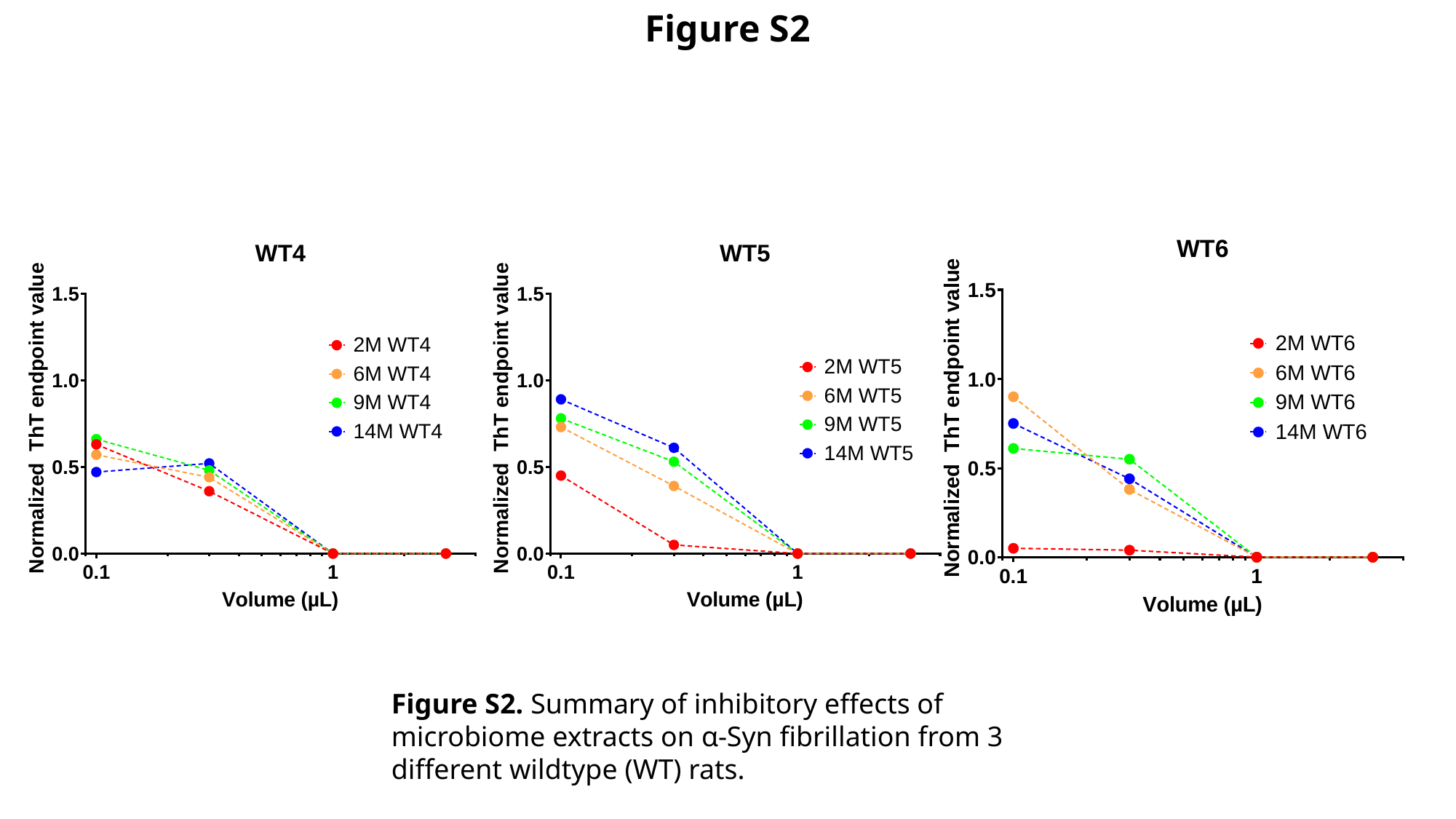

Figure S2
Figure S2. Summary of inhibitory effects of microbiome extracts on α-Syn fibrillation from 3 different wildtype (WT) rats.

## Slide 4
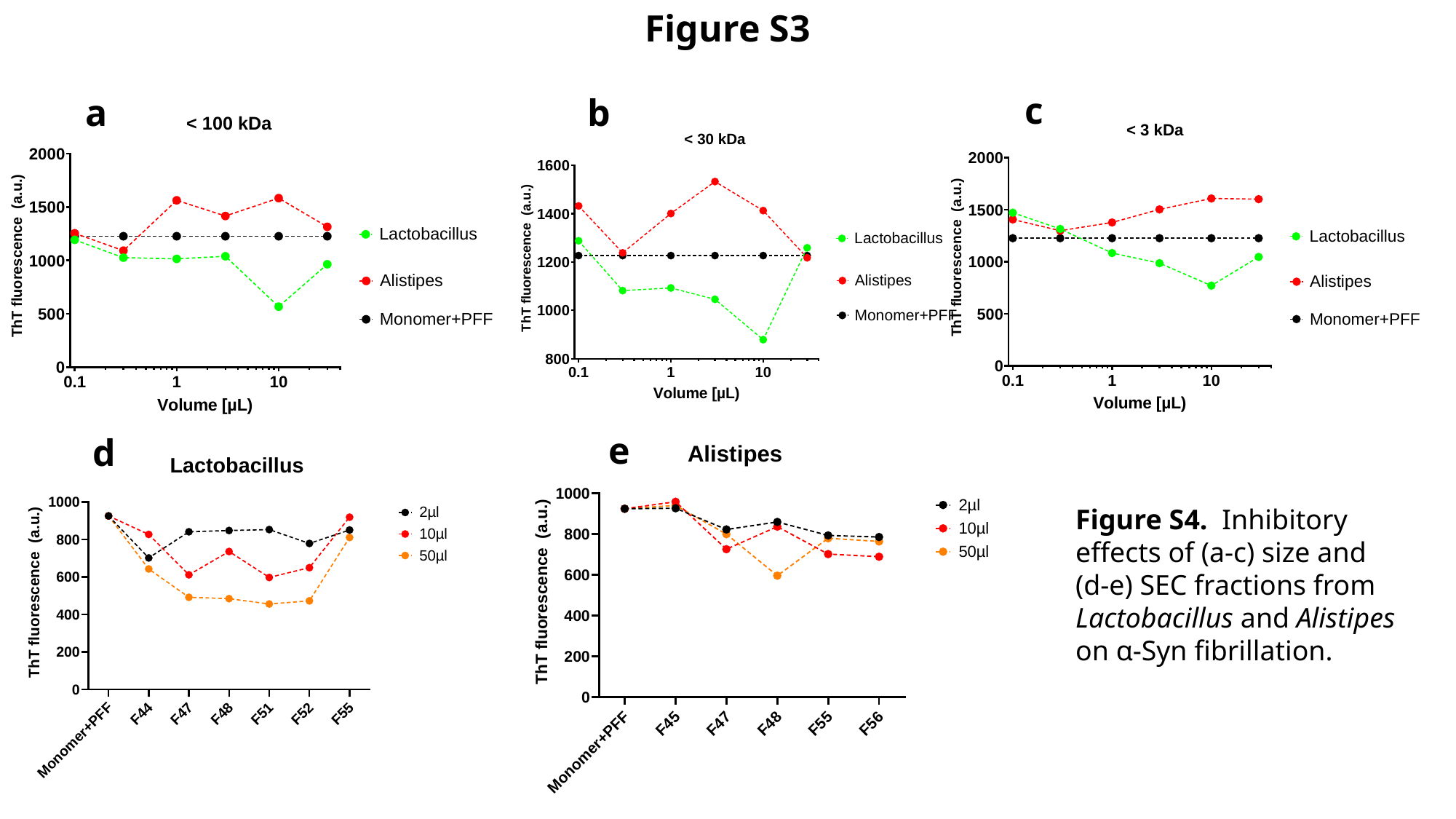

Figure S3
c
a
b
e
d
Figure S4. Inhibitory effects of (a-c) size and (d-e) SEC fractions from Lactobacillus and Alistipes on α-Syn fibrillation.

## Slide 5
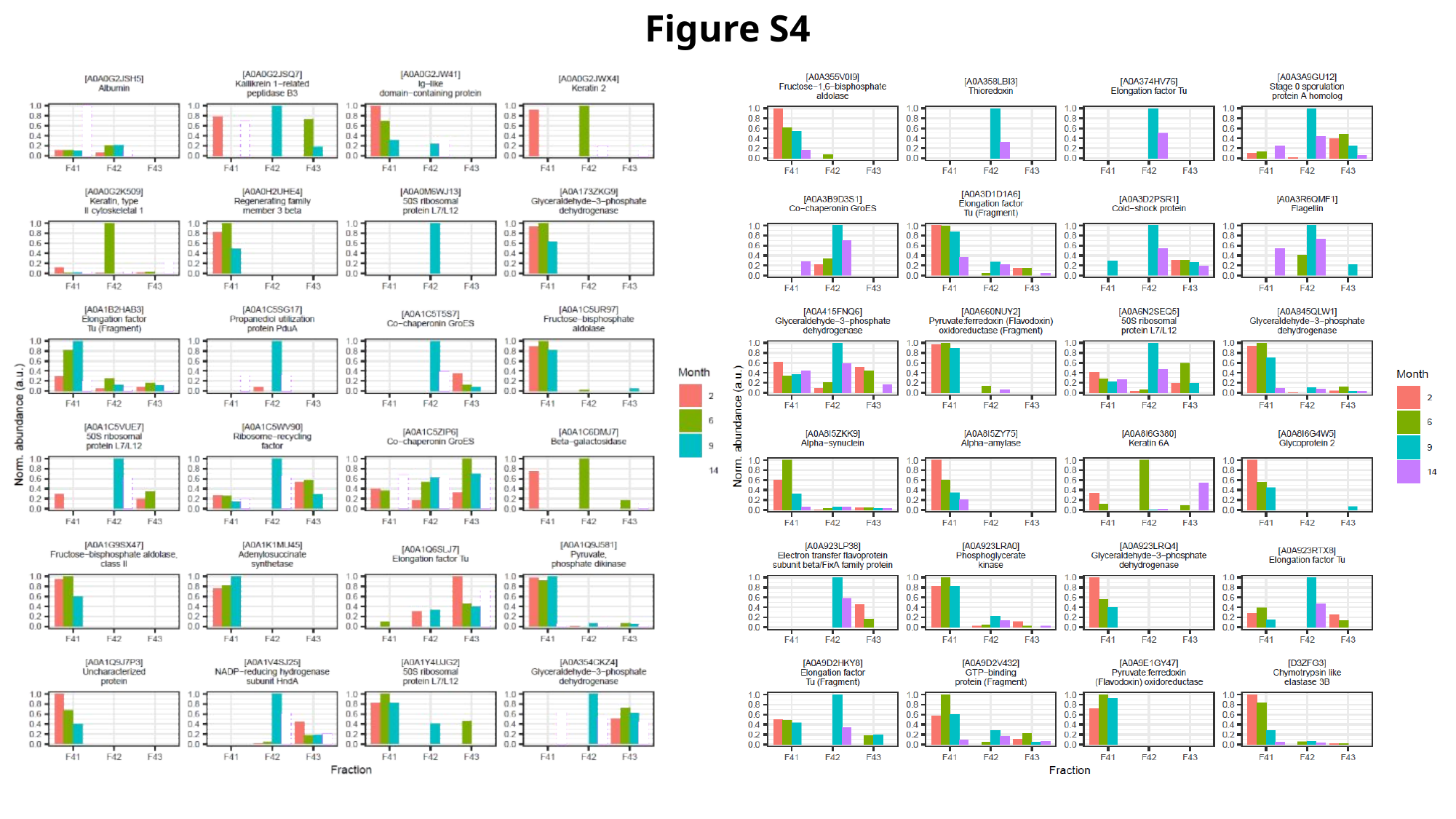

Figure S4

## Slide 6
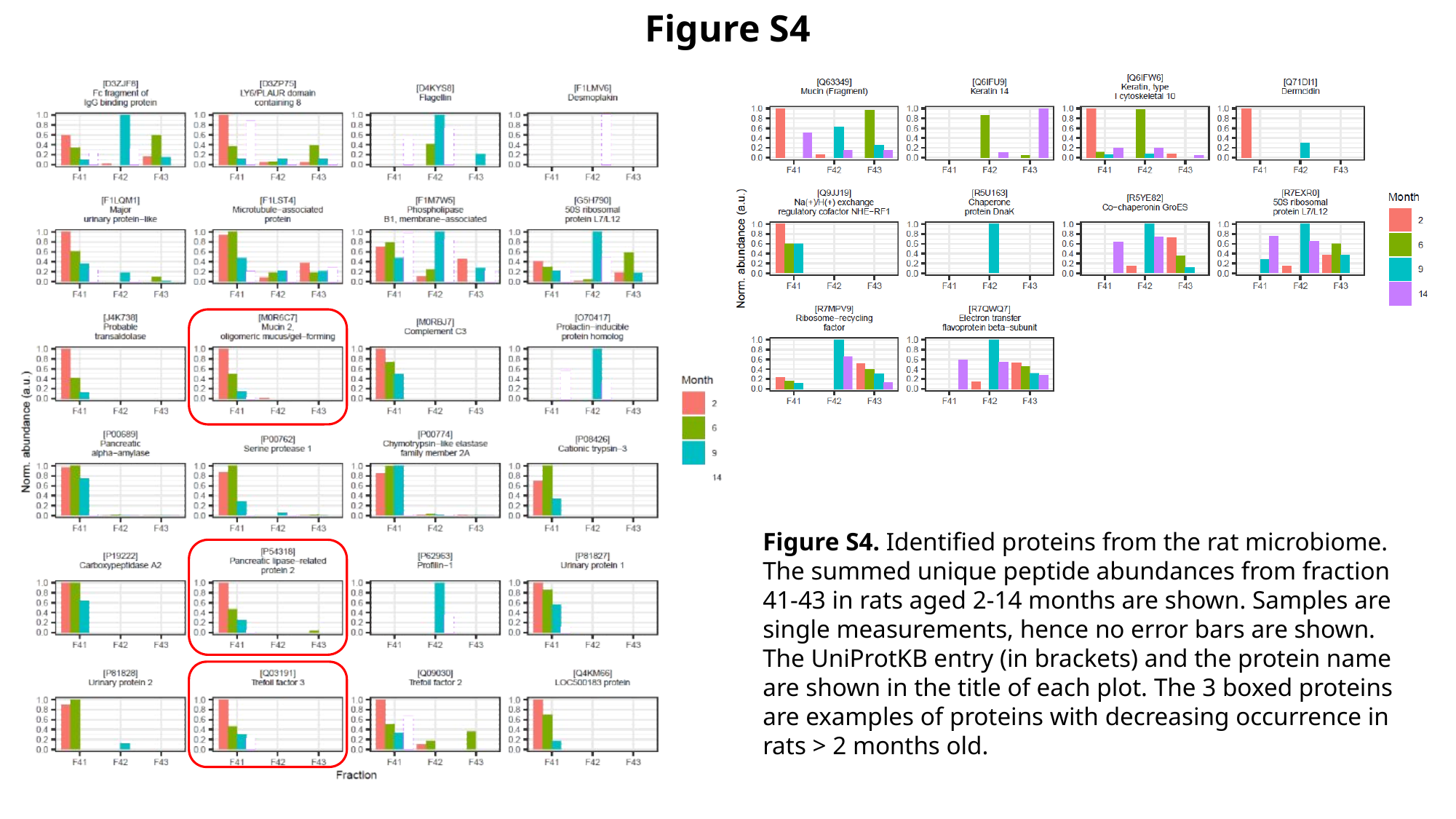

Figure S4
Figure S4. Identified proteins from the rat microbiome. The summed unique peptide abundances from fraction 41-43 in rats aged 2-14 months are shown. Samples are single measurements, hence no error bars are shown. The UniProtKB entry (in brackets) and the protein name are shown in the title of each plot. The 3 boxed proteins are examples of proteins with decreasing occurrence in rats > 2 months old.

## Slide 7
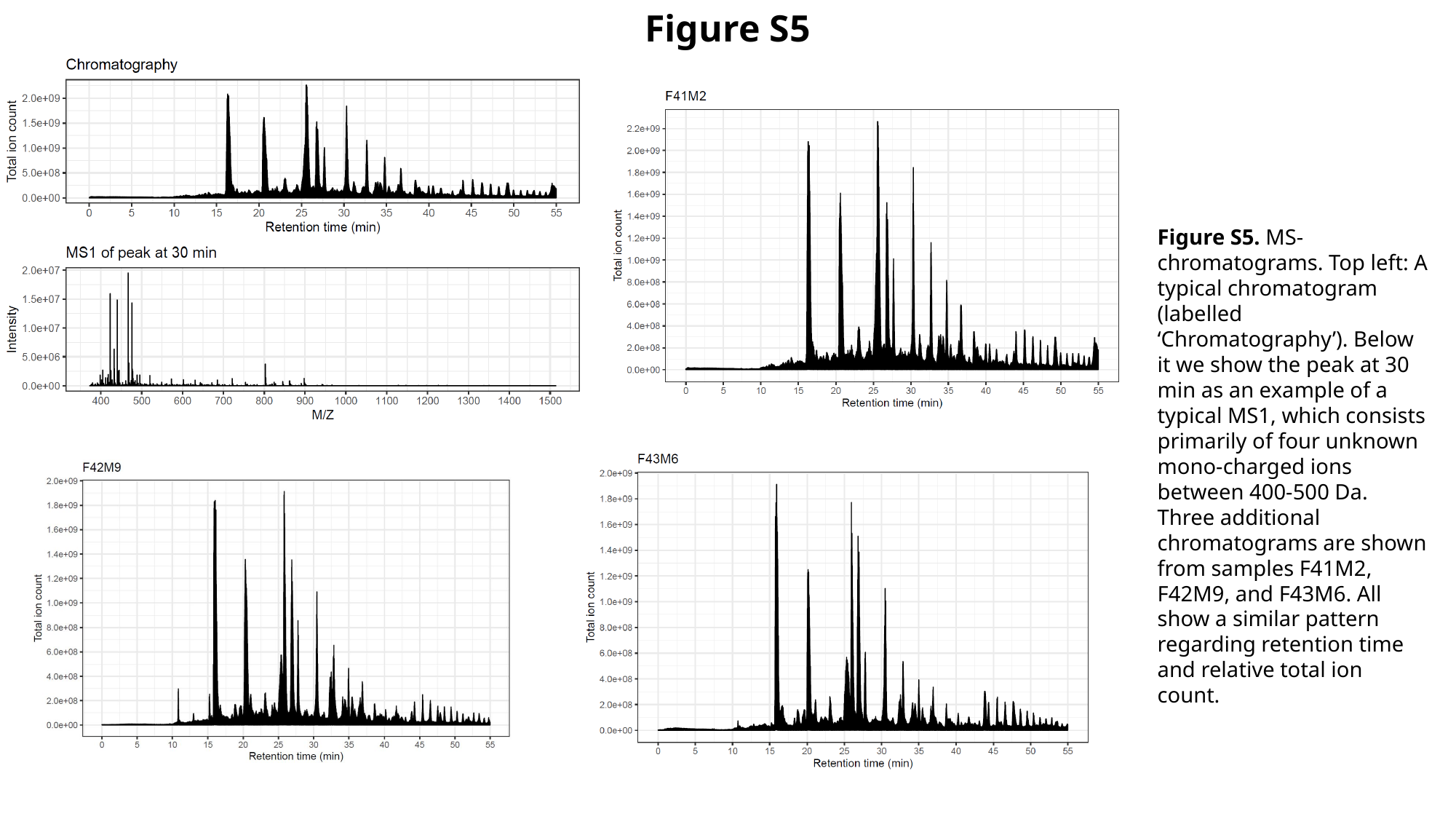

Figure S5
Figure S5. MS-chromatograms. Top left: A typical chromatogram (labelled ‘Chromatography’). Below it we show the peak at 30 min as an example of a typical MS1, which consists primarily of four unknown mono-charged ions between 400-500 Da. Three additional chromatograms are shown from samples F41M2, F42M9, and F43M6. All show a similar pattern regarding retention time and relative total ion count.

## Slide 8
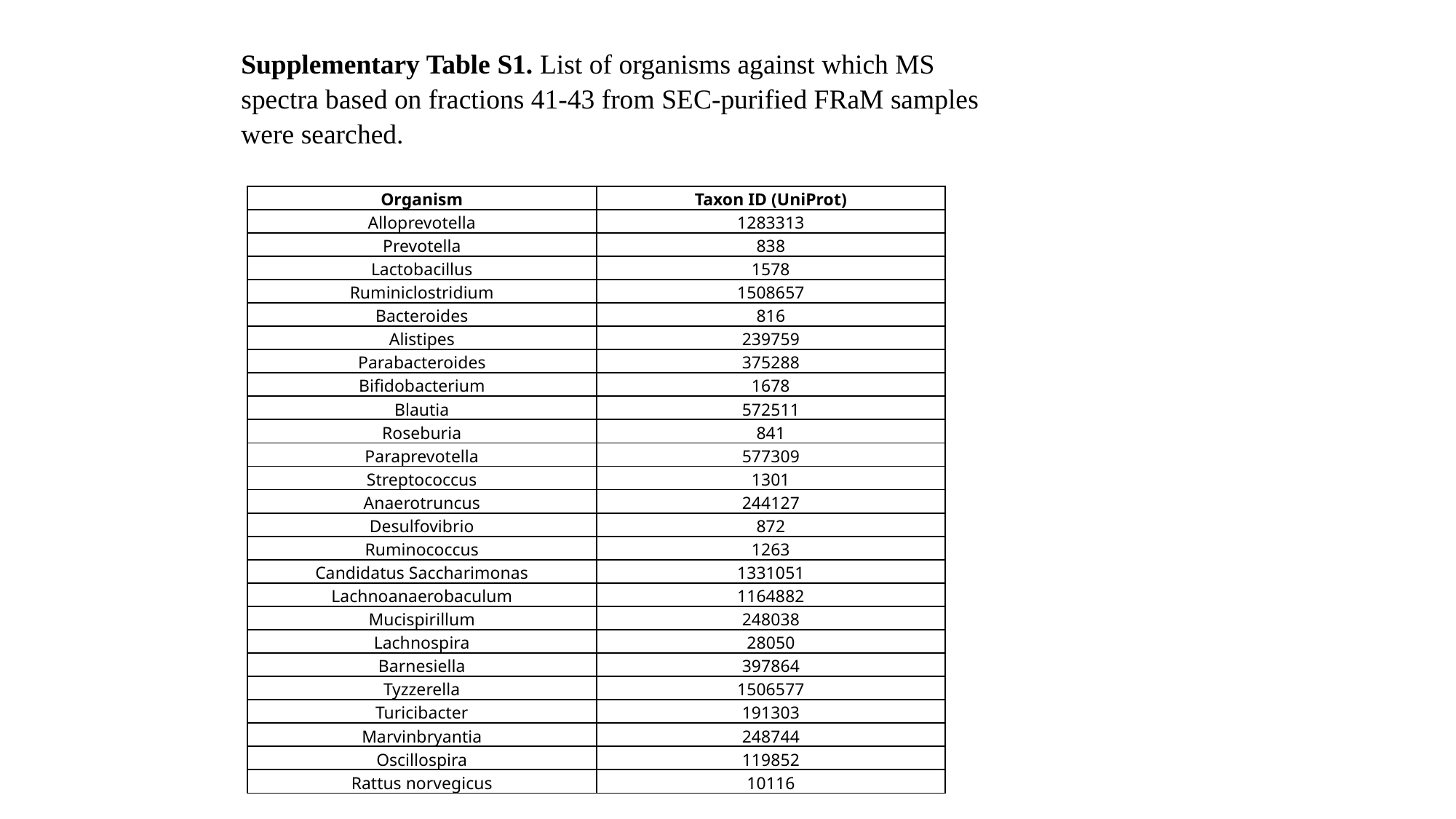

Supplementary Table S1. List of organisms against which MS spectra based on fractions 41-43 from SEC-purified FRaM samples were searched.
| Organism | Taxon ID (UniProt) |
| --- | --- |
| Alloprevotella | 1283313 |
| Prevotella | 838 |
| Lactobacillus | 1578 |
| Ruminiclostridium | 1508657 |
| Bacteroides | 816 |
| Alistipes | 239759 |
| Parabacteroides | 375288 |
| Bifidobacterium | 1678 |
| Blautia | 572511 |
| Roseburia | 841 |
| Paraprevotella | 577309 |
| Streptococcus | 1301 |
| Anaerotruncus | 244127 |
| Desulfovibrio | 872 |
| Ruminococcus | 1263 |
| Candidatus Saccharimonas | 1331051 |
| Lachnoanaerobaculum | 1164882 |
| Mucispirillum | 248038 |
| Lachnospira | 28050 |
| Barnesiella | 397864 |
| Tyzzerella | 1506577 |
| Turicibacter | 191303 |
| Marvinbryantia | 248744 |
| Oscillospira | 119852 |
| Rattus norvegicus | 10116 |
